# MicroRNAs from edible plants reach the human gastrointestinal tract and may act as potential regulators of gene expression

**DOI:** 10.1007/s13105-024-01023-0

**Published:** 2024-04-25

**Authors:** Ester Díez-Sainz, Fermín I. Milagro, Paula Aranaz, José I. Riezu-Boj, Silvia Lorente-Cebrián

**Affiliations:** 1 Department of Nutrition, Food Science and Physiology/Center for Nutrition Research, Faculty of Pharmacy and Nutrition, University of Navarra, 31008 Pamplona, Spain; 2 Navarra Institute for Health Research (IdiSNA), 31008 Pamplona, Spain; 3 Centro de Investigación Biomédica en Red Fisiopatología de La Obesidad y Nutrición (CIBERobn), Instituto de Salud Carlos III, 28029 Madrid, Spain; 4 Department of Pharmacology, Physiology and Legal and Forensic Medicine, Faculty of Health and Sport Science, University of Zaragoza, 50009 Saragossa, Spain; 5 Instituto Agroalimentario de Aragón-IA2, Universidad de Zaragoza-CITA, 50013 Saragossa, Spain; 6 Aragón Health Research Institute (IIS-Aragon), 50009 Saragossa, Spain

**Keywords:** Cross-kingdom regulation, XenomiRs, Plant miRNAs, Diet

## Abstract

MicroRNAs (miRNAs) are small single-stranded non-coding RNA molecules that regulate gene expression at the post-transcriptional level. A cross-kingdom regulatory function has been unveiled for plant miRNAs (xenomiRs), which could shape inter-species interactions of plants with other organisms (bacteria and humans) and thus, be key functional molecules of plant-based food in mammals. However, discrepancies regarding the stability and bioavailability of dietary plant miRNAs on the host cast in doubt whether these molecules could have a significant impact on human physiology. The aim of the present study was to identify miRNAs in edible plants and determine their bioavailability on humans after an acute intake of plant-based products. It was found that plant food, including fruits, vegetables and greens, nuts, legumes, and cereals, contains a wide range of miRNAs. XenomiRs miR156e, miR159 and miR162 were detected in great abundance in edible plants and were present among many plant foods, and thus, they were selected as candidates to analyse their bioavailability in humans. These plant miRNAs resisted cooking processes (heat-treatments) and their relative presence increased in faeces after and acute intake of plant-based foods, although they were not detected in serum. Bioinformatic analysis revealed that these miRNAs could potentially target human and bacterial genes involved in processes such as cell signalling and metabolism. In conclusion, edible plants contain miRNAs, such as miR156e, miR159 and miR162, that could resist degradation during cooking and digestion and reach the distal segments of the gastrointestinal tract. Nevertheless, strategies should be developed to improve their absorption to potentially reach host tissues and organs and modulate human physiology.

**Supplementary Information:**

The online version contains supplementary material available at 10.1007/s13105-024-01023-0.

## Introduction

MicroRNAs (miRNAs) are non-coding RNAs of ~ 22 nucleotides that are master regulators of gene expression at the post-transcriptional level [8]. In plants, miRNAs play part in biological functions, such as growth and development, stress responses and metabolism [22, 67]. Remarkably, a key role in cross-kingdom communication have been unveiled for plant miRNAs, which could modulate the interactions of plants with microorganisms and animals [35]. Plant miRNAs can be up-taken by pathogens inhibiting their virulence, and by intestinal bacteria thus having an impact on gut microbiota composition, localization, and metabolite production [58, 70]. Plant miRNAs can also interact with mammalian cells and exert several biological functions, including anti-viral, anti-tumour, immuno-modulatory and anti-inflammatory as well as anti-apoptotic effects [35]. In fact, it has been suggested that the use of plant miRNAs as therapeutic agents could be an effective strategy to treat a wide variety of diseases, such as cancer, COVID-19 and chronic-inflammatory diseases [11, 42, 55, 58, 59]. The effect of plants miRNAs in human gene expression and biological functions has been studied in vitro. For instance, plant miRNAs could exert anti-inflammatory effects through the modulation of *CLEC7A*, *NFAM1* genes or by direct binding to TLR3 receptor in immune system cells [11, 20]. In addition, plant miRNAs promote anti-tumoral responses by enhancing apoptosis and suppressing proliferation of human tumour cells [42, 47]. Plant miRNAs could also regulate the expression of metabolic genes, such as *LDLRAP1* (intestinal cells), *QKI* and *MK2* (hepatocytes) and decrease lipid accumulation in hepatocytes [19, 69].

Diet would be the main source of plant miRNAs in mammals. However, it is still unclear whether dietary miRNAs could reach host cells and display a significant biological impact, by which plant miRNA could act as cross-kingdom gene expression regulators. Plant miRNAs have been found in urine, faeces, serum and tissues of mammals such as mice, pigs and humans [12, 37, 39, 40, 62, 65, 66, 69, 72]. In addition, gene expression and physiological changes in animals, such the improvement of metabolic parameters or the decrease of inflammation, have been detected upon the intake of exogenous plant miRNAs (plant xenomiRs) [4, 59, 69]. However, these results are not supported by other authors, who reported negligible levels of xenomiRs on animals, suggesting that the delivery of dietary plant miRNAs on the host would be ineffective [18, 46, 63].

It is well known that plant foods possess beneficial properties that could be used in the management of human diseases, such as obesity, non-alcoholic fatty liver disease, respiratory diseases, cancer, diabetes, anxiety and depression [7, 16, 27, 32, 48, 60]. We hypothesize that the beneficial properties of plants in human physiology could partially rely on cross-kingdom communication through specific plant miRNAs. For this reason, it is important to determine (1) if miRNAs could be relevant effector molecules of the therapeutic effects of plants in humans, and (2) if dietary intake of plants could provide sufficient bioavailability of miRNAs on the host, to be considered as bioactive ingredients in humans. According to this, in the present study, we aimed to identify miRNAs from edible plants and to determine their presence in the human gut and circulatory system upon an acute intake of different plant foods. Moreover, we selected some of the most abundant and conserved plant miRNAs and performed a bioinformatic analysis of their putative biological effects on human cells and bacterial cells.

## Materials and Methods

### RNA isolation from edible plants, human serum and faecal samples

Total RNA was extracted from cereals (rice), vegetables and greens (green beans, green peppers, lettuces, and spinaches), fruits (apples, olives, oranges, pears, and tomatoes), legumes (chickpeas and lentils), and nuts (walnuts), using miRNeasy Serum/Plasma Kit (Qiagen, Hilden, Germany), according to manufacturer’s instructions. Briefly, raw plants products were used, except for green beans, legumes, and cereals: green beans, lentils and chickpeas were boiled for 4.5, 15 and 30 min, respectively, in a pressure cooker (chickpeas and lentils were soaked previously during 12 h and 2 h, respectively). Rice was boiled for 20 min using a casserole. 0.2 g of cereals, 0.1 g of vegetables and greens, fruits and legumes, and 0.05 g of nuts, were grounded in 1 ml of QIAzol Lysis Reagent with Ultra-Turrax T25 Basic (Basic IKA- Werke, Staufen, Germany) for 1 min (vegetables and greens, fruits, and nuts) or 2 min (cereals and legumes), on ice and at the highest speed. Samples were centrifuged at 12,000 g at 4 ºC for 15 min (olive, vegetables and greens) or 5 min (fruits except olive, cereals, legumes, and nuts). Olive samples formed a fat layer, which was removed, and samples were centrifuged at 12,000 g at 4 ºC for 5 min. The supernatants from plant samples were used for RNA isolation. Prior to the RNA extraction of plant samples used for quantitative real-time PCR (qPCR) assays, 1 µl of spike-in controls (RNA Spike-In Kit, For RT. Qiagen).

Plant miRNA expression analyses in humans were performed as a proof-of-concept study from anonymous healthy volunteers (> 18 yr.). They had a two-day wash out period with poor vegetable intake and then increased their plant intake for three-days. Specifically, volunteers followed an enriched plant-based diet which included several groups of plant foods, such as nuts, legumes, fruits, vegetables and cereals, in the proportion and variety they preferred. Faecal and serum samples were collected before and after the time course; no personal data were obtained from these subjects. Faeces were collected in stool nucleic acid collection and preservation tubes (Norgen Biotek Corp., ON, Canada), and 250 µl of sample was used to isolate total RNA with RNeasy PowerMicrobiome Kit (Qiagen). Venous blood was collected in BD Vacutainer® SST™ II Advance tubes (BD Vacutainer Systems, Plymouth, United Kingdom), sera was separated by centrifugation at 16,000 g at 4 ºC for 10 min, and 200 µl of sample was used to isolate total RNA with miRNeasy Serum/Plasma Advanced Kit (Qiagen). Prior to the extraction, 1 µl of spike-in controls was added to each serum and faecal sample according to manufacturer’s instructions.

### Identification of miRNAs in plant samples by next-generation sequencing

Next generation sequencing (NGS) analyses were conducted with total RNA from plants samples, including vegetables (spinach), nuts (walnut) and fruits (apple, olive, pear, orange, and tomato), which were selected after performing quality controls. Library preparation and amplification was carried out using NEBNext® Small RNA Library Prep Set for Illumina® kit and NEBNext® Multiplex Oligos for Illumina (Index Primers Set 1‐4) (New England Biolabs, Ipswich, MA, USA), following manufacturer’s instructions. Removal of big fragment was achieved by bead-based purification as follows: samples were incubated twice with AgenCourt AMPure XP beads (Beckman Coulter, Brea, CA, USA), firstly at a ratio of 1.3x, and secondly at a ratio of 3.7x. Size distribution of the libraries was estimated using Agilent Bioanalyzer (Agilent Technologies, Santa Clara, CA, USA). Quantification of final libraries was carried out by qPCR using the KAPA Library Quantification Kit (KAPA Biosystems Inc., Wilmington, MA, USA), prior to the amplification with Illumina's cBot. Libraries were sequenced 1 × 50 + 8 bp on Illumina's HiSeq2500. Output raw data were processed with skewer [29] to remove the adapter, and reads (from 15 to 30 bp) were aligned with a plant reference genome (*Prunus persica*, NCBIv2 and annotation NCBIv2.52 restricted to miRNAs) (https://www.ncbi.nlm.nih.gov/assembly/GCF_000346465.2/#/def_asm_Primary_Assembly), with maximum 2 mismatches in the seed area, using miRNA annotation from miRbase (version 22) and ShortStack [5]. Htseq-count [3] was used to count mapped tags, considering the strand information. Raw reads were normalized with DESeq2. Sequencing data were deposited in NCBI's Gene Expression Omnibus [23], and are accessible through GEO Series accession number GSE234786 (https://www.ncbi.nlm.nih.gov/geo/query/acc.cgi?acc=GSE234786).

### Plant-derived miR156e, miR159 and miR162 expression analysis

The expression of the xenomiRs miR156e, miR159 and miR162 (identified by NGS) was analysed by qPCR in RNA from: cereals (cooked rice), vegetables and greens (green peppers, lettuces, spinaches, raw and cooked green beans), fruits (apples, olives, oranges, pears, tomatoes), cooked legumes (chickpeas and lentils), nuts (walnuts), and human samples (serum and faeces). The expression of the exogenous spike-in UniSp4 was used as a positive control in plant and human samples to determine the efficiency of the experimental procedure. In addition, in human samples, the expression of the endogenous hsa-miR-141-3p and hsa-miR-103a-3p was analysed to check the sample quality.

Reverse-transcription was performed with 4 µl of total RNA with miRCURY LNA RT Kit (Qiagen), in a final volume reaction of 10 µl. Reactions were conducted at 42 °C for 60 min and 95 ºC for 5 min, in a MyCycler Thermal Cycler (Bio-Rad, Hercules, CA, USA). cDNA from plant samples was centrifuged at 4 ºC for 1 min at the highest speed to collect the supernatant for qPCR. cDNA (diluted 1/10) was amplified with miRCURY LNA miRNA PCR Assays (Table 1) and miRCURY LNA SYBR Green PCR Kit (Qiagen), in a CFX384 Touch Real-Time PCR detection System (Bio-Rad). Control samples (non-template) were added to each reaction. The cycling conditions were 95 ºC for 2 min, 40 cycles at 95 ºC for 10 s and 56 ºC for 1 min. miRNA expression levels (Cq values) in serum and faecal samples were normalized with the spike-in UniSp4 (ΔCq) and the following formula was applied to compare the number of copies of plant miRNAs before and after the acute intake of plant foods: 2 ^(ΔCq before plant acute intake – ΔCq after plant acute intake)^.

**Table 1:** Assays used for quantitative PCR

miRNA name	miRBase accession number	Assay reference (GenGlobe ID)	Assay sequence
ppe-miR156e	MIMAT0031432	YP02102391	5'-UGACAGAAGAGAGUGAGCAC-3'
ppe-miR159	MIMAT0031437	YP02100546	5'-UUUGGAUUGAAGGGAGCUCUA-3'
ppe-miR162	MIMAT0031440	YP02106385	5'-UCGAUAAACCUCUGCAUCCAG-3'
hsa-miR-141-3p	MIMAT0000432	YP00204504	5'-UAACACUGUCUGGUAAAGAUGG-3'
hsa-miR-103a-3p	MIMAT0000101	YP00204063	5'-AGCAGCAUUGUACAGGGCUAUGA-3'
UniSp4	N/A	YP00203953	N/A

N/A: not applicable

### Bioinformatic analyses to predict potential human and bacterial targets of plant miRNAs

The small RNA target analysis servers TAPIR (https://www.zhaolab.org/psRNATarget/) [9] and psRNATarget (scoring schemas V1 and V2) (https://bioinformatics.psb.ugent.be/webtools/tapir/) [15] were used to identify putative human target genes of miR156e (5'-UGACAGAAGAGAGUGAGCAC-3'), miR159 (5'-UUUGGAUUGAAGGGAGCUCUA-3') and miR162 (5'-UCGAUAAACCUCUGCAUCCAG-3'). Plant miRNA sequences were aligned to the cDNA library “*Homo sapiens* (human), transcript, Human genomic sequencing project” (available in the psRNATarget server), applying the default parameters. The online tools Genecodis (https://genecodis.genyo.es/) [10] and PANTHER (https://pantherdb.org/) [45] were used to conduct Gene Ontology (GO) enrichment analysis and biological process classification. In addition, pathway analyses were performed with Genecodis, by which annotations from different sources were used (KEGG Pathways, Panther Pathways and WikiPathways). These analyses were carried out independently for each miRNA, with the putative target genes identified with TAPIR and psRNATarget. A cut-off threshold for the expectation value, which represents the penalty for the mismatches between miRNA and target sequence, was applied to filtered psRNATarget scoring schema V2 putative targets: the cut-off value was set between 3.5–4.5 in order to select up to 20 top target genes. For PANTHER analyses (access on 26 May 2023) “*Homo sapiens*” was selected as reference organism.

Prediction analyses of bacterial targets of plants miRNAs were conducted with TargetRNA3 (https://cs.wellesley.edu/~btjaden/TargetRNA3/), applying the default parameters [61]. Ten genomes from eight different bacteria species were selected *Escherichia coli* (*Escherichia coli* ATCC 25922 (GCF_017357505.1); *Escherichia coli* str. K-12 substr. MG1655 (GCF_000005845.2); *Escherichia coli* O157:H7 str. Sakai Sakai substr. RIMD 0509952 (GCF_000008865.2)), *Enterococcus faecalis* (*Enterococcus faecalis* OG1RF (GCF_000172575.2)), *Bifidobacterium longum* (*Bifidobacterium longum* subsp. longum JCM 1217 (GCF_000196555.1)), *Lactobacillus acidophilus* (*Lactobacillus acidophilus* La-14 (GCF_000389675.2)), *Levilactobacillus brevis* (*Levilactobacillus brevis* NPS-QW-145 (GCF_001676805.1)), *Limosilactobacillus fermentum* (*Limosilactobacillus fermentum* SCB0035 (GCF_022819245.1)), *Ligilactobacillus salivarius* (*Ligilactobacillus salivarius* LPM01 (GCF_900094615.1)), *Lactobacillus jensenii* (*Lactobacillus jensenii* SNUV360 (GCF_001936235.1)).

### Statistical analysis

Wilcoxon matched-pairs signed rank test was applied to determine differences in human samples before and after the acute intake of plant food products. Differences were considered statistically significant at p-value *p* < 0.05.

## Results

### miRNAs diversity and abundance in edible plants

The miRNA profile was analysed by NGS in the following edible plants: fruits (apple, olive, orange pear and tomato), vegetables (spinach), and nuts (walnut): They were selected because of their very good results in the quality controls (bioanalyzer determinations). The results revealed that 176 miRNAs were present in at least one of the plants used for this study. miR156e (gene:ENSRNA049996234), miR159 (gene:ENSRNA049996936), and miR162 (gene:ENSRNA049996910) were selected as candidate miRNAs for subsequent analyses due to their broad presence among the selected plant species and high abundance (number of reads) (Table S1).

**Fig. 1:**
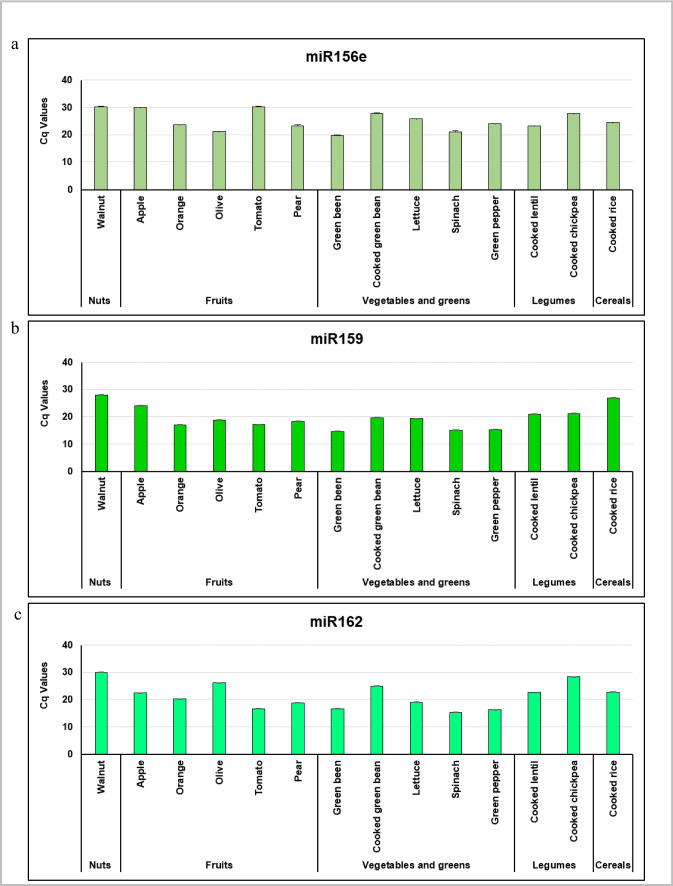
Detection of miR156e, miR159 and miR162 in raw and cooked edible plant products by quantitative PCR. The results are presented as the Cq values of (**a**) miR156e (5'-UGACAGAAGAGAGUGAGCAC-3'), (**b**) miR159 (5'-UUUGGAUUGAAGGGAGCUCUA-3'), and (**c**) miR162 (5'-UCGAUAAACCUCUGCAUCCAG-3') in fruits (apple, orange, olive, tomato, and pear), vegetables and greens (raw and cooked green beans, lettuce, spinach and green pepper), nuts (walnuts), cooked legumes (lentil and chickpea) and cooked cereals (rice). Results are expressed as the mean ± standard error of the mean (SEM) (*n* = 2)

The expression of miR156e, miR159 and miR162 in edible plants was validated by qPCR in an extended selection of raw and cooked food matrices: nuts (walnuts), fruits (apple, orange, olive, tomato, and pear), vegetables and greens (raw and cooked green beans, lettuce, spinach and green pepper), cooked legumes (lentils and chickpeas) and cooked cereals (rice). The three plant miRNAs were present in all the plant foods analysed (Fig. 1). Notably, the three miRNAs were also present in boiled green beans, lentils, chickpeas, and rice, which is the process that they usually undergo before being consumed. Since the objective was to identify miRNAs that were present in the plants, rather than quantifying miRNAs and comparing their abundance between different samples, data were not normalized. Nonetheless, spike-in UniSp4 was added prior to the RNA isolation and whose detection was used as a positive (reference) control to determine the reliability of the results.

### Plant xenomiRs miR156e, miR159 and miR162 expression is increased in human faecal samples after an acute intake of plant-origin foods

To determine the presence of plant miRNAs in human samples, seven healthy volunteers were subjected to a three days-acute intake of a wide variety of plant products (see MM). The expression profile of miR156e, miR159 and miR162 was analysed in faecal and serum samples before and after the intervention. The expression levels of two endogenous human miRNAs, hsa-miR-141-3p and hsa-miR-103a-3p, were also evaluated in faecal and serum samples, respectively to determine sample quality and data reliability. Providing that no universal endogenous miRNA normalizer (housekeeping) has been standardized yet, exogenous spike-in UniSp4 expression was used to eliminate variability of RNA extraction, reverse-transcription, and qPCR processes.

**Fig. 2:**
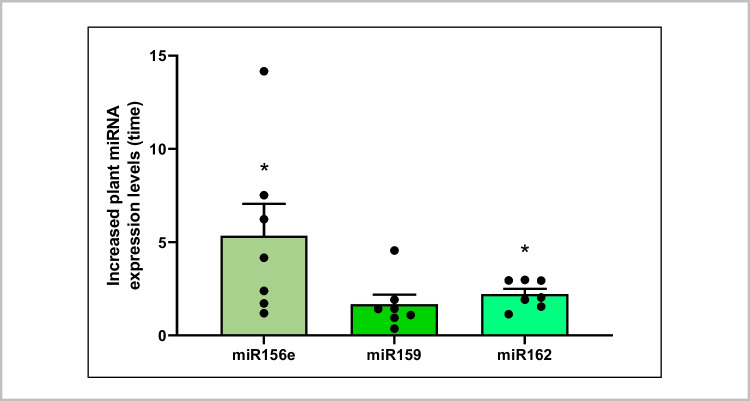
Relative quantification of plant miRNAs miR156e, miR159 and miR162 in human faeces after a three-day time course acute intake of plant products. Plant miRNAs miR156e, miR159 and miR162 expression was analysed by qPCR in human faecal samples from 7 volunteers before and after a dietary intervention consisting of a high consumption of plant products for three days. Cq values were normalized (ΔCq) with the spike-in UniSp4 and expression level differences before and after the interventions were expressed as 2 ^(ΔCq before plant acute intake – ΔCq after plant acute intake)^. Results are presented as the mean ± standard error of the mean (SEM) (*n* = 7 volunteers). *p*-value: * *p* < 0.05 as compared vs. before the acute intake

Plant miR156e, miR159 and miR162 were present in all the faecal samples and their expression levels increased 5.34 ± 1.72 (*p* < 0.05), 1.68 ± 0.51 (ns), and 2.21 ± 0.28 (*p* < 0.05) times, respectively, after the high intake of plant foods as compared to their respective levels at baseline (Fig. 2). Undetectable levels of plant miRNAs were reported in serum samples of the same set of individuals, suggesting that the selected plant miRNAs were not absorbed. However, the endogenous control hsa-miR-103a-3p was amplified (Cq values between 24–28). This demonstrates that human-origin miRNAs are present in serum samples while (exogenous) plant xenomiRs are not present or are available in such low levels that are far beyond qPCR detection limit.

### Plant miRNAs miR156e, miR159 and miR162 could potentially target human and bacterial genes and modulate host biological functions and pathways

To evaluate if plant miR156e, miR159 and miR162 could exert biological effects on human cells, bioinformatic analyses were carried out to potentially verify plant miRNA impact on shaping host gene expression. miR156e, miR159 and miR162 mature sequences were aligned with the human transcriptome to predict putative human target genes using psRNATarget (scoring schemas V1 and V2) and TAPIR algorithms. miR156e could potentially modulate the expression of 166 different transcripts (152 different human genes) (Table S2). Of note, the *F11R* gene (transcript NM_016946) appeared in the three prediction algorithms (Table S2). In addition, the three algorithms predicted that a total of 132 different transcripts (120 different genes) could be potentially regulated by miR159, of which the transcript NM_005444 (*RQCD1* gene) was a common output (Table S3). psRNATarget scoring schema V2 was the only algorithm that reported putative targets (40 different transcripts, 37 different genes) for miR162 (Table S4).

To identify the human biological functions and pathways that could be modulated by the predicted targets of miR156e, miR159 and miR162, we conducted Gene Ontology (GO) and KEGG, Panther and WikiPathways analyses:

- For miR156e putative targes, 19 genes were analysed (*HIF3A*, *F11R*, *SUCLG2*, *ALG2*, *SV2B*, *C12orf74*, *KCTD18*, *HORMAD2*, *EHMT1*, *FHL1*, *ANKRD13A, LPGAT1*, *VSX1*, *ARF3, ARL4C*, *POLR3H*, *SEC23IP*, *CCDC88C*, *ZFP62*). In PANTHER software, one gene was not detected (*C12orf74*) and ten genes were unclassified (*ALG2*, *HORMAD2*, *FHL1*, *EHMT1*, *ZFP62*, *SV2B*, *LPGAT1*, *ANKRD13A*, *SEC23IP*, *KCTD18*). In KEGG Pathways, Panther Pathways and WikiPathways, the genes that were reported as unannotated inputs were: *ANKRD13A*, *KCTD18*, *SEC23IP*, *VSX1*, *ZFP62*, *C12orf74* (KEGG, Panther and Wiki Pathways), *ARL4C*, *CCDC88C*, *HIF3A*, *HORMAD2* (KEGG and Panther Pathways), *SV2B* (Panther and Wiki Pathways), *F11R*, *SUCLG2*, *ALG2*, *EHMT1*, *FHL1*, *LPGAT1*, *POLR3H* (Panther Pathways), *ARF3* (Wiki Pathways).
- For miR159, 14 genes were analysed (*AMOT*, *RQCD1*, *TRIM14*, *RBAK*, *PPM1E*, *RGAG4*, *AK1*, *LPP*, *IRS1*, *ENKUR*, *PIM3*, *RIC3*, *SEH1L*, *STON1*). In PANTHER, one gene was not detected (*RGAG4*), two were unclassified (*ENKUR*, *TRIM14*). In GO analysis with Genecodis, *RGAG4* was also an unannotated input. In KEGG Pathways, Panther Pathways and WikiPathways, the genes that were reported as unannotated inputs were: *ENKUR*, *PIM3*, *PPM1E*, *RIC3*, *RGAG4*, *STON1*, *TRIM14* (KEGG, Panther and Wiki Pathways), *LPP* (KEGG and Panther Pathways), *RBAK*, *SHE1L* (Panther and Wiki Pathways), *AMOT*, *RQCD1* (Panther Pathways), *AK1* (Wiki Pathways).
- For miR162, seven genes were introduced (*MAPK14*, *CISD3*, *RAB3D*, *PLG*, *PRR5L*, *DNM2*, *RBAK*). In PANTHER analyses, two were unclassified (*DNM2*, *CISD3*). In KEGG Pathways, Panther Pathways and WikiPathways, the genes that were reported unannotated as inputs were: *CISD3* (KEGG, Panther and Wiki Pathways), *PRR5L* (KEGG and Panther Pathways), *RAB3D*, *RBAK* (Panther and Wiki Pathways), *DNM2* (Panther Pathways).

**Fig. 3:**
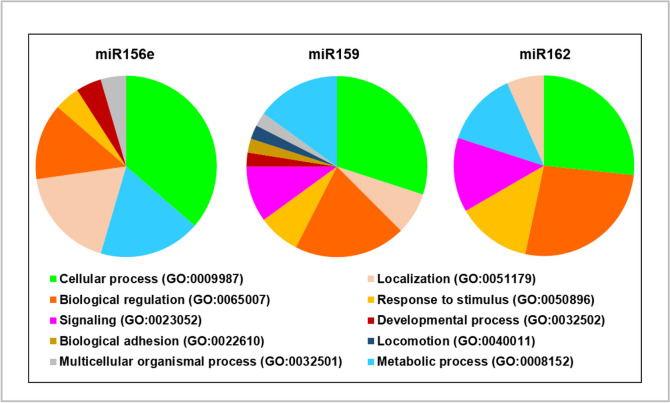
Gene ontology analysis of predicted target genes of plant miRNAs, miR156e, miR159 and miR162, performed by PANTHER. Putative target identified with TAPIR and psRNATarget scoring schemas V1 and V2 were classified based on biological process annotation. Results are presented in pie charts, filtering genes with no PANTHER category assigned

In terms of biological process, 7, 10 and 6 groups were identified by PANTHER for miR156e, miR159 and miR162 predicted target genes, respectively, of which “cellular process” was the top group (Fig. 3). Predicted targets were also clustered in other groups, including “cell signalling” (miR159 and miR162 putative targets), “metabolic process” and “response to stimulus” (all three). GO results performed with Genecodis showed that miR156e putative targets are enriched in biological processes that include stress-activated protein kinase signalling cascade (*CCDC88C*), memory T cell extravasation, and regulation of membrane permeability and bicellular tight junction assembly (*F11R*), (Table 2), and the Huntington disease pathway (*ARL4C* and *ARF3*) (Table 5). Predicted target genes of plant miR159 were enriched in processes such as negative regulation of insulin secretion involved in cellular response to glucose stimulus (*PIM3*), positive regulation of fatty acid beta-oxidation and glucose metabolism process (*IRS1*) (Table 3), and pathways such as insulin signalling and type II diabetes mellitus (*IRS1*) (Table 5). The biological functions of miR162 target genes are gathered in Table 4, and include micropinocytosis, positive regulation of endocytosis (*DNM2*), stress-induced premature senescence, regulation of cytokine production involved in inflammatory response, fatty acid oxidation and response to dietary excess (*MAPK14*), while no biological pathways were identified with a significant adjusted *p* value (Table 5).

**Table 2:** Gene Ontology (GO) Biological processes of the putative human target genes of miR156e, performed with Genecodis

Plant miR156e putative target genes		
Biological Process	Relative enrichment	Genes
Protein Glycosylation In Endoplasmic Reticulum (GO:0033577)	473.76	*ALG2*
Oligosaccharide-Lipid Intermediate Biosynthetic Process (GO:0006490)	236.88	
Negative Regulation Of Protein Localization To Endosome (GO:1905667)	947.53	*ANKRD13A*
Apical Constriction (GO:0003383)	236.88	*CCDC88C*
Stress-Activated Protein Kinase Signalling Cascade (GO:0031098)	236.88	
Histone H3-K27 Methylation (GO:0070734)	236.88	*EHMT1*
Peptidyl-Lysine Dimethylation (GO:0018027)	135.36	
Memory T Cell Extravasation (GO:0035683)	473.76	*F11R*
Protein Localization To Bicellular Tight Junction (GO:1902396)	236.88	
Regulation Of Membrane Permeability (GO:0090559)	157.92	
Positive Regulation Of Establishment Of Endothelial Barrier (GO:1903142)	157.92	
Regulation Of Bicellular Tight Junction Assembly (GO:2000810)	105.28	
Regulation Of Potassium Ion Transmembrane Transporter Activity (GO:1901016)	157.92	*FHL1*
Meiotic Sister Chromatid Cohesion (GO:0051177)	189.51	*HORMAD2*
Phosphatidylinositol Acyl-Chain Remodelling (GO:0036149)	157.92	*LPGAT1*
Transcription Initiation From RNA Polymerase III Promoter (GO:0006384)	135.36	*POLR3H*
Succinyl-Coa Metabolic Process (GO:0006104)	189.51	*SUCLG2*
Retinal Bipolar Neuron Differentiation (GO:0060040)	315.84	*VSX1*

Putative targets identified with TAPIR and psRNATarget scoring schema V1 and V2, were used. The table shows biological processes of miR156e putative target genes with an adjusted *p* value ≤ 0.05 and a relative enrichment ≥ 100

**Table 3:** Gene Ontology (GO) Biological processes of the putative human target genes of miR159, performed with Genecodis

Plant miR159 putative target genes		
Biological Process	Relative enrichment	Genes
ADP Biosynthetic Process (GO:0006172)	276.97	*AK1*
AMP Metabolic Process (GO:0046033)	197.84	
Nucleobase-Containing Small Molecule Interconversion (GO:0015949)	173.11	
Nucleoside Triphosphate Biosynthetic Process (GO:0009142)	138.48	
Nucleoside Monophosphate Phosphorylation (GO:0046940)	125.90	
Establishment Of Cell Polarity Involved In Ameboidal Cell Migration (GO:0003365)	461.62	*AMOT*
Cell Migration Involved In Gastrulation (GO:0042074)	153.87	
Positive Regulation Of Cell Size (GO:0045793)	138.48	
Blood Vessel Endothelial Cell Migration (GO:0043534)	138.48	
Establishment Of Left/Right Asymmetry (GO:0061966)	276.97	*ENKUR*
Positive Regulation Of Glucose Metabolic Process (GO:0010907)	138.48	*IRS1*
Positive Regulation Of Fatty Acid Beta-Oxidation (GO:0032000)	125.90	
Negative Regulation Of Insulin Secretion Involved In Cellular Response To Glucose Stimulus (GO:0061179)	197.84	*PIM3*
Nuclear Pore Organization (GO:0006999)	173.11	*SEH1L*
Attachment Of Mitotic Spindle Microtubules To Kinetochore (GO:0051315)	125.90	

Putative targets identified with TAPIR and psRNATarget scoring schema V1 and V2, were used. The table shows biological processes of miR159 putative target genes with an adjusted *p* value ≤ 0.05 and a relative enrichment ≥ 100

**Table 4:** Gene Ontology (GO) Biological processes of the putative human target genes of miR162, performed with Genecodis

Plant miR162 putative target genes		
Biological Process	Relative enrichment	Genes
Protein Maturation By [2Fe-2S] Cluster Transfer (GO:0106034)	1285.93	*CISD3*
Cellular Response To Carbon Monoxide (GO:0071245)	2571.86	*DNM2*
Negative Regulation Of Membrane Tubulation (GO:1903526)	1285.93	
Positive Regulation Of P-Type Sodium:Potassium-Exchanging Transporter Activity (GO:1903408)	857.29	
Negative Regulation Of Non-Motile Cilium Assembly (GO:1902856)	857.29	
Positive Regulation Of Clathrin-Dependent Endocytosis (GO:2000370)	514.37	
Macropinocytosis (GO:0044351)	321.48	
Transferrin Transport (GO:0033572)	257.19	
Cellular Response To X-Ray (GO:0071481)	233.81	
G Protein-Coupled Receptor Internalization (GO:0002031)	214.32	
Regulation Of Rac Protein Signal Transduction (GO:0035020)	197.84	
Regulation Of Axon Extension (GO:0030516)	183.70	
Regulation Of Golgi Organization (GO:1903358)	183.70	
Synaptic Vesicle Transport (GO:0048489)	171.46	
Cellular Response To Dopamine (GO:1903351)	160.74	
Cellular Response To Nitric Oxide (GO:0071732)	151.29	
Post-Golgi Vesicle-Mediated Transport (GO:0006892)	128.59	
Positive Regulation Of Endocytosis (GO:0045807)	111.82	
Response To Light Stimulus (GO:0009416)	107.16	
Golgi To Plasma Membrane Transport (GO:0006893)	102.87	
Positive Regulation Of Cyclase Activity (GO:0031281)	857.29	*MAPK14*
Stress-Induced Premature Senescence (GO:0090400)	642.96	
Regulation Of Synaptic Membrane Adhesion (GO:0099179)	642.96	
Signal Transduction In Response To DNA Damage (GO:0042770)	257.19	
Positive Regulation Of Muscle Cell Differentiation (GO:0051149)	257.19	
Regulation Of Cytokine Production Involved In Inflammatory Response (GO:1900015)	233.81	
Cellular Response To Lipoteichoic Acid (GO:0071223)	233.81	
Response To Muramyl Dipeptide (GO:0032495)	214.32	
P38Mapk Cascade (GO:0038066)	214.32	
Negative Regulation Of Hippo Signalling (GO:0035331)	197.84	
Response To Dietary Excess (GO:0002021)	183.70	
Regulation Of Ossification (GO:0030278)	171.46	
Fatty Acid Oxidation (GO:0019395)	171.46	
Positive Regulation Of Myotube Differentiation (GO:0010831)	171.46	
Positive Regulation Of Myoblast Fusion (GO:1901741)	160.74	
Positive Regulation Of Brown Fat Cell Differentiation (GO:0090336)	160.74	
Striated Muscle Cell Differentiation (GO:0051146)	160.74	
Transmembrane Receptor Protein Serine/Threonine Kinase Signalling Pathway (GO:0007178)	142.88	
Response To Muscle Stretch (GO:0035994)	142.88	
3'-Utr-Mediated mRNA Stabilization (GO:0070935)	128.59	
Cartilage Condensation (GO:0001502)	128.59	
Positive Regulation Of Myoblast Differentiation (GO:0045663)	102.87	
Positive Regulation Of Blood Vessel Endothelial Cell Migration (GO:0043536)	102.87	*MAPK14, PLG*
Mononuclear Cell Migration (GO:0071674)	1285.93	*PLG*
Trans-Synaptic Signalling By Bdnf, Modulating Synaptic Transmission (GO:0099183)	642.96	
Positive Regulation Of Fibrinolysis (GO:0051919)	642.96	
Biological Process Involved In Interaction With Symbiont (GO:0051702)	514.37	
Negative Regulation Of Cell–Cell Adhesion Mediated By Cadherin (GO:2000048)	257.19	
Tissue Remodelling (GO:0048771)	257.19	
Negative Regulation Of Fibrinolysis (GO:0051918)	257.19	
Trophoblast Giant Cell Differentiation (GO:0060707)	171.46	
Myoblast Differentiation (GO:0045445)	151.29	
Negative Regulation Of Cell-Substrate Adhesion (GO:0010812)	142.88	
Fibrinolysis (GO:0042730)	142.88	
Labyrinthine Layer Blood Vessel Development (GO:0060716)	128.59	
Muscle Cell Cellular Homeostasis (GO:0046716)	122.47	
Tissue Regeneration (GO:0042246)	111.82	
Torc2 Signalling (GO:0038203)	367.41	*PRR5L*
Regulation Of Fibroblast Migration (GO:0010762)	214.32	
Positive Regulation Of mRNA Catabolic Process (GO:0061014)	171.46	
Peptidyl-Cysteine Methylation (GO:0018125)	1285.93	*RAB3D*
Positive Regulation Of Regulated Secretory Pathway (GO:1903307)	514.37	
Bone Resorption (GO:0045453)	107.16	

Putative targets identified with TAPIR and psRNATarget scoring schema V1 and V2, were used. The table shows biological processes of miR162 putative target genes with an adjusted *p* value ≤ 0.05 and a relative enrichment ≥ 100

**Table 5:** Biological pathway analyses of the putative human target genes of miR156e and miR159 performed with Genecodis

Plant miR156e putative target genes		
**Panther Pathways**	**Relative enrichment**	**Genes**
Huntington disease (P00029)	17.67	*ARL4C, ARF3*
**Plant miR159 putative target genes**		
**WikiPathways**	**Relative enrichment**	**Genes**
Insulin signalling in adipocytes (normal condition) (WP3634)	243.19	*IRS1*
Insulin signalling in adipocytes (diabetic condition) (WP3635)	243.19	
Leptin-insulin signalling overlap (WP3935)	114.44	
Congenital generalized lipodystrophy (CGL) (WP5101)	102.39	
FOXA2 pathway (WP5066)	92.64	
Transcription factor regulation in adipogenesis (WP3599)	88.43	
Type II diabetes mellitus (WP1584)	88.43	
EPO receptor signalling (WP581)	74.83	
MFAP5 effect on permeability and motility of endothelial cells via cytoskeleton rearrangement (WP4560)	108.08	*LPP*

Putative targets identified with TAPIR and psRNATarget scoring schema V1 and V2, were used. The table shows biological pathways of miR156e and miR159 putative target genes with an adjusted *p* value ≤ 0.05

To determine if plant miR156e, miR159 and miR162 could potentially have an impact on (gut-present) bacteria, eight bacteria species that have been found in the human gut were selected (*Escherichia coli*, *Enterococcus faecalis* and several species of *Lactobacillus*) [17, 26, 41, 44, 52, 54, 57, 73]. Bioinformatic analyses were conducted with TargetRNA3 algorithm to predict targets in prokaryotes (Table 6). miR156e could potentially modulate the expression of two different targets in *Escherichia Coli*: *fadK* (a short chain acyl-CoA synthetase) and the hypothetical protein ECs_5262. Moreover, *MukB* (a chromosome partitioning protein) and the hypothetical protein ECs_5262 from *Escherichia Coli*, and *pknB* (a Stk1 family PASTA domain-containing Ser/Thr kinase) from *Ligilactobacillus salivarius*, were predicted as targets of miR159. Finally, several putative targets of miR162 were identified: *cysI* (a sulfite reductase subunit) of *Escherichia coli*, OG1RF_RS11400 (ribonuclease J) of *Enterococcus faecalis*, BLLJ_RS06465 (ABC transporter ATP-binding protein) of *Bifidobacterium longum*, and *ppx* (exopolyphosphatase) of *Levilactobacillus brevis*.

**Table 6:** Bioinformatic analysis to predict putative bacteria targets of plant miR156e, miR159 and miR162 performed by TargetRNA3 software

Plant microRNA	Bacteria specie	Target	Annotation	*p*-value	Probability*
miR156e	*Escherichia coli* str. K-12 substr. MG1655 (GCF_000005845.2)	*fadK* (b1701)	Short chain acyl-CoA synthetase	2.20E-06	0.51
	*Escherichia coli* O157:H7 str. Sakai Sakai substr. RIMD 0509952 (GCF_000008865.2)	ECs_5262	Hypothetical protein	4.57E-07	0.70
miR159	*Escherichia coli* str. K-12 substr. MG1655 (GCF_000005845.2)	*mukB* (b0924)	Chromosome partitioning protein MukB	1.98E-06	0.59
	*Escherichia coli* O157:H7 str. Sakai Sakai substr. RIMD 0509952 (GCF_000008865.2)	ECs_5262	Hypothetical protein	8.1253E-07	0.70
		*mukB* (ECs_1007)	Chromosome condensin MukBEF ATPase and DNA-binding subunit MukB	3.4152E-06	0.59
	*Escherichia coli* ATCC 25922 (GCF_017357505.1)	*mukB* (D1792_RS23855)	Chromosome partition protein MukB	3.74E-06	0.59
	*Ligilactobacillus salivarius* LPM01 (GCF_900094615.1)	*pknB* (BQ1177_RS07590)	Stk1 family PASTA domain-containing Ser/Thr kinase	2.21E-05	0.51
miR162	*Escherichia coli* str. K-12 substr. MG1655 (GCF_000005845.2)	*cysI* (b2763)	Sulfite reductase, hemoprotein subunit	8.36E-07	0.52
	*Escherichia coli* O157:H7 str. Sakai Sakai substr. RIMD 0509952 (GCF_000008865.2)	*cysI* (ECs_3618)	Sulfite reductase beta subunit	7.78E-07	0.52
	*Enterococcus faecalis* OG1RF (GCF_000172575.2)	OG1RF_RS11400	Ribonuclease J	3.23E-06	0.51
	*Bifidobacterium longum* subsp. *longum* JCM 1217 (GCF_000196555.1)	BLLJ_RS06465	ABC transporter ATP-binding protein	1.77E-06	0.52
	*Levilactobacillus brevis* NPS-QW-145 (GCF_001676805.1)	*ppx* (A6F53_RS04985)	Exopolyphosphatase	3.27E-07	0.54

Bacteria genomes were aligned with miR156e (5'-UGACAGAAGAGAGUGAGCAC-3'), miR159 (5'-UUUGGAUUGAAGGGAGCUCUA-3') and miR162 (5'-UCGAUAAACCUCUGCAUCCAG-3') mature sequences. *Probability refers to the possibility that there is a regulatory interaction between the small RNA and the predicted target. Probability greater than 0.5 indicates that the candidate is more likely to be a target of the small RNA than not to be one

## Discussion

This study contributed to the identification of the miRNA expression profile of edible plants. Small-RNA sequencing results revealed that plant-based diets could be a major source of plant xenomiRs. miR156e, miR159 and miR162 were selected as candidates for downstream analysis due to their abundance and high conservation degree across the selection of analysed plant foods. However, this selection does not exclude the possibility that other identified miRNAs could be interesting research elements for new investigations. Notably, these miRNAs were present in plant food products (legumes, cereals, and green beans) after boiling, suggesting that extreme processes do not jeopardise the stability (and presumably functionality) of plant miRNAs. These results are in agreement with other studies reporting that miR166, miR167 and miR168 from soybean and rice resisted storage, processing and cooking conditions [51], and miR156a, miR166a and miR168a were also stable in cooked rice [69]. Indeed, results from the present study might suggest that miR156e, miR159 and miR162 could be good candidates to generally estimate the bioavailability of plant miRNAs in humans, since they were present in great amount in all groups of plant foods tested (legumes, nuts, fruits, cereals, vegetables and greens) and they resisted physical treatments (soaking and heat-treatments). Of note, no universal plant miRNA housekeeping has been identified to potentially quantify the relative expression of target xenomiRs. We evaluated miRNA levels as a proof-of-concept (validation) of their presence in different food matrices (qualitative value). Therefore, we consider that normalizing the relative miRNA expression is not necessary for the specific aim in the framework of the present study (we did not want to quantify, just qualitatively demonstrate their abundance: present/not present).

Plant miR156e, miR159 and miR162 were detected in faeces of healthy volunteers, and the relative abundance of plant miR156e and miR162 increased after a three-day acute intake of plant products. These results revealed that plant miRNAs could reach the gastrointestinal tract resisting the digestive processes. In addition, it was unveiled that dietary modifications such as increasing plant intake enhance exogenous miRNA bioavailability. Importantly, efforts should not be focused exclusively on the evaluation of plant miRNA effects in distal tissues and organs and on developing strategies to eventually promote their absorption, since their biological impact could be exerted and limited to at local-gut level. Several published studies support this hypothesis. For instance, Teng et al. reported that ginger EVs can be taken by gut microorganisms and their miRNA cargo modulates bacteria gene expression, shaping immune system responses and improving gut barrier function [58]. In this study, we demonstrate that plant miR156e, miR159 and miR162 reach the human gastrointestinal tract in agreement with previous studies, which showed that miR156 could reach mice gut and regulate enterocyte growth [36]. In concordance with the studies that unveiled a crosstalk between plant miRNAs mdo-miR7267-3p and bol-miR159 and bacteria which shaped metabolite production, composition, localization, and growth of gut microbes [58, 64], we conducted bioinformatic analyses to eventually identify non-human (prokaryote) targets of plant miRNAs. Our results revealed a potential interaction of plant miR156e, miR159 and miR162 and bacterial targets. Thus, miR156e, miR159, and miR162 could potentially modulate the expression of targets involved in bacterial metabolism (i.e., *fadK* and *cysI*), bacteria growth (i.e., *mukB*), and stress responses (i.e., *ppx*), and thus, could contribute to gut eubiosis and subsequent impact in host physiology [6, 24, 33, 49]. In addition, we performed bioinformatic predictions, GO and pathways analysis to predict putative human target genes of plant miR156e, miR159 and miR162 and biological functions. The data presented here suggest that miR156 could potentially modulate the expression of human genes involved in intestinal homeostasis, such as the establishment of the endothelial intestinal barrier, in agreement with the results of Li et al. [36]. Importantly, Li et al. also showed that the administration of synthetic miR156 could modulate mammalian enterocyte growth by targeting *WNT10B *in vitro and in mice [36]. Our in silico analyses show that this effect in intestinal homeostasis could potentially be achieved through the modulation of *F11R* gene, which appeared in all the prediction programs used (psRNATarget scoring schema V1 and V2 and TAPIR). Knockdown of *F11R* gene (which encodes for JAM-A protein) has been previously associated with the leaky gut and gut barrier disruption [14, 53]. By contrast, positive effects have been achieved though *F11R* downregulation, which comprise counteraction of cancer progression [34, 68]. However, the potential biological impact of bioinformatics predictions in this article is still to be fully determined.

Nonetheless, plant miRNAs were undetectable in serum samples of the same volunteers. Our data suggest that plant miRNA absorption and distribution to peripheral organs and tissues cannot be achieved by implementing dietary modifications (increase of plant food intake) or under our experimental conditions. These results do not support the observation made by other authors, which found that plant miRNAs could be detected in the circulatory system of a diverse type of animals, such as mice, pigs and humans, and be distributed to tissues and organs [37, 38, 40, 65, 66, 69]. Interestingly, Chen et al. analysed blood samples from NGS datasets and detected differences in the expression profile of plant miRNAs in animals with different dietary regimes, determining that plant miRNA abundance in blood was higher in herbivores, followed by omnivorous and they were barely detected in carnivores [12]. However, absorption of plant miRNAs in animals has been questioned by several reports, which claimed that detection of plant miRNAs might be artifacts of human sequence contaminations during NGS [25, 30, 62, 71]. In this context, the results presented in this article are consistent with the observations made by other studies where absorption of plant miRNAs could be undetected or even inexistent. Increasing plant intake, such pollen, corn, rice or extra virgin olive oil or fruits, in a wide range of animals, including honey bees, mice and humans, reported no measurable levels of plants miRNAs in blood or tissues [18, 28, 43, 46, 56]. The discrepancies concerning the bioavailability of plant miRNAs in animals may be explained by different factors: (1) plant miRNA administration methods (i.e., oral intake, gavage, administration of isolated RNA or plant-based products), dosage/amount administered and exposure time; (2) sensibility of the miRNA detection techniques; (3) gut permeability; and (4) miRNA stability (each plant miRNA could have different availability depending on its physical–chemical stability, and eventually protective factors like encapsulation in extracellular vesicles (EVs) [21, 65, 66]. As we report here, plant miRNAs are not detected in blood despite increasing plant intake.

Notably, our bioinformatic analyses suggest that plant miRNAs could not only have a potential impact at the local-gut level, but also at peripheral tissues and organs. miR159 could potentially modulate the expression of genes related with metabolic pathways (i.e., glucose metabolism and insulin signalling pathways) and immune system. Moreover, *RQCD1* could be a putative target gene of miR159; it appeared in all the prediction algorithms and its upregulation has been linked to breast cancer progression [1]. Notably, functional validation of miR159 interactions with mammalian genes, that were not identified in this work using TAPIR and psRNATarget has been already unveiled [4, 13]. Aquilano et al. [4] reported that mimics for plant miR159 could target *Tnfrsf1a* gene in obese mice, supressing inflammation and improving the metabolic profile. Chin et al. demonstrated that miR159 targets *TCF7* gene and its administration counteracted xenograft breast tumour growth in mice [13]. miR156e could also have an impact beyond the gut level. For instance, GO and pathways analyses unveiled and association between its predicted human targets and neuron differentiation and Huntington disease. In this context, in silico analyses have already revealed the therapeutic potential of plants miRNAs to treat neurological disorders, such as Alzheimer [50]. However, the results of the present work suggest that plant miRNAs might not surpass the gut barrier, since they were not detected in serum samples, at least in the conditions of our study. Despite these results, we hypothesize that other (dietary) strategies could be addressed to potentially promote the absorption of miRNAs and enhance their potential therapeutic effects in vivo. In this context, miRNA stability and bioavailability could be improved by encapsulation in EVs. Notably, plant EVs have been postulated as promising therapeutic delivery systems, which confer protection to their cargo, can be internalized by mammalian cells, are stable in several conditions, such as storage and digestion, and their internal content is highly bioavailable [2, 31]. Therefore, the administration of nanocarriers designed to contain cocktails of plant miRNAs with therapeutic benefits could be worthwhile to promote their absorption since the evidence presented here unveil that they were not present in the circulatory system, but they could potentially exert a biological impact beyond the gut.

In conclusion, the present article has contributed to the identification of miRNA expression profile in plant foods, revealing that edible plant-based products contain large number of miRNAs, which can resist cooking processes. In addition, it has been unveiled that a short plant-based dietary intervention can increase the abundance of exogenous miRNAs in the gut. Therefore, significant effects of plant miRNAs on human physiology could potentially be displayed thought the interaction with gut cells (i.e. enterocytes) and bacteria. Nevertheless, the negligible levels of plant miRNAs detected in serum highlight the need to develop novel strategies to improve the bioavailability of plant miRNAs beyond the gut. For instance, the administration of nanocarriers designed to contain cocktails of plant miRNAs with therapeutic benefits could be worthwhile. In any case, our data suggest that miR156e, miR159 and miR162 have the potential to affect the expression of human and bacterial genes and modulate important biological pathways.

## Supplementary Information

Below is the link to the electronic supplementary material.

Supplementary file1 (DOCX 152 KB)

## Data Availability

The data supporting the conclusions of this work are available by the authors on request. Next generation sequencing data are publicly available at https://www.ncbi.nlm.nih.gov/geo/query/acc.cgi?acc=GSE234786.
