# Supplementary material for: MicroRNAs from edible plants reach the human gastrointestinal tract and may act as potential regulators of gene expression
Source: J Physiol Biochem. 2024 Apr 25;80(3):655–70. doi: 10.1007/s13105-024-01023-0 (PMC11502557; doi:10.1007/s13105-024-01023-0)
Supplement: Supplementary file 1 — Supplementary file1 (DOCX 152 KB) [file 13105_2024_1023_MOESM1_ESM.docx]

**MicroRNAs from edible plants reach the human gastrointestinal tract and may act as potential regulators of gene expression**

Ester Díez-Sainz ^1^, Fermín I. Milagro ^1,2,3^*, Paula Aranaz ^1,2^, José I. Riezu-Boj ^1,2^, Silvia Lorente-Cebrián ^4,5,6^

^1^ Department of Nutrition, Food Science and Physiology/Center for Nutrition Research, Faculty of Pharmacy and Nutrition, University of Navarra, 31008 Pamplona, Spain; ediezsainz@alumni.unav.es (E.D.); fmilagro@unav.es (F.M.); paranaz@unav.es (P.A.); jiriezu@unav.es (J.R.)

^2^ Navarra Institute for Health Research (IdiSNA), 31008 Pamplona, Spain

^3^ Centro de Investigación Biomédica en Red Fisiopatología de la Obesidad y Nutrición (CIBERobn), Instituto de Salud Carlos III, 28029 Madrid, Spain

^4^ Department of Pharmacology, Physiology and Legal and Forensic Medicine, Faculty of Health and Sport Science, University of Zaragoza, 50009 Zaragoza, Spain.; slorentec@unizar.es (S.L.)

^5^ Instituto Agroalimentario de Aragón-IA2, Universidad de Zaragoza-CITA, 50013 Zaragoza, Spain

^6^ Aragón Health Research Institute (IIS-Aragon), 50009 Zaragoza, Spain

***** Correspondence: fmilagro@unav.es // https://orcid.org/0000-0002-3228-9916

**Supplementary material**

**Table S1. Identification of miR156e, miR159 and miR159 in plant foods by next-generation sequencing.**

| **Gene ID** | **miRNA name** | **Normalized reads** | | | | | | |
| --- | --- | --- | --- | --- | --- | --- | --- | --- |
|  |  | **Walnut** | **Apple** | **Olive** | **Orange** | **Pear** | **Tomato** | **Spinach** |
| gene:ENSRNA049996234 | miR156e | 43.29 | 2.05 | 10.25 | 8.57 | 65.73 | 0.00 | 48.92 |
| gene:ENSRNA049996936 | miR159 | 2747.28 | 75.07 | 972.57 | 2908.03 | 355.22 | 304.59 | 972.57 |
| gene:ENSRNA049996910 | miR162 | 230.03 | 300.31 | 0.68 | 71.37 | 1697.31 | 582.74 | 213.56 |

The results are expressed as the normalized reads of a single sample per plant product.

**Table S2. Bioinformatic analysis to predict putative human target genes of plant miR156e performed by psRNATarget (scoring schemas V1 and V2) and TAPIR software.**

| **Plant miR156e putative target genes** | | | | |
| --- | --- | --- | --- | --- |
| **psRNATarget. Scoring Schema V1** | | | | |
| **Target accession** | **Expectation** | **UPE** | **mRNA target aligned fragment (5’-3’)** | **Inhibitory effect** |
| NM_152795\|*HIF3A* | 3 | 21.17 | 3601-[CUGCUCGCUCUGUUUUGUCA]-3620 | Translation |
| **NM_016946\|*F11R*** | **3** | **23.039** | **504-[GGGCCCACUCUCUUCUGUCU]-523** | **Cleavage** |
| NM_003848\|*SUCLG2* | 3 | 20.101 | 210-[GUUCUCAUACUUUUCUGUCA]-229 | Cleavage |
| NM_033087\|*ALG2* | 3 | 15.213 | 432-[GUGUUCACUGUCAUCUGUUA]-451 | Translation |
| NM_014848\|*SV2B* | 3 | 15.484 | 1791-[AUGUUUACUCUCCUUCUGUCA]-1811 | Cleavage |
| **psRNATarget. Scoring Schema V2** | | | | |
| **Target accession** | **Expectation** | **UPE** | **mRNA target aligned fragment (5’-3’)** | **Inhibitory effect** |
| NM_001178097\|*C12orf74* | 2.5 | N/A | 190-[UCGCUCACUUUCUUCUGUCC]-209 | Cleavage |
| NM_152795\|*HIF3A* | 2.5 | N/A | 3601-[CUGCUCGCUCUGUUUUGUCA]-3620 | Cleavage |
| NM_152387\|*KCTD18* | 2.5 | N/A | 602-[UUUCUUACUCUUUUCUGUUA]-621 | Cleavage |
| NM_152510\|*HORMAD2* | 3 | N/A | 115-[UUGCUCAAUUUUUUUUGUCA]-134 | Cleavage |
| NM_024757\|*EHMT1* | 3 | N/A | 1060-[AUGUUUAUUUUUUUCUGUUA]-1079 | Cleavage |
| NM_001167819\|*FHL1* | 3 | N/A | 429-[CUGCUCACCCUCUUCUGUGA]-448 | Cleavage |
| NM_001159703\|*FHL1* | 3 | N/A | 500-[CUGCUCACCCUCUUCUGUGA]-519 | Cleavage |
| **NM_016946\|*F11R*** | **3** | **N/A** | **504-[GGGCCCACUCUCUUCUGUCU]-523** | **Cleavage** |
| NM_033121\|*ANKRD13A* | 3 | N/A | 1204-[UAGUUAACUCUCUUUUGUCA]-1223 | Cleavage |
| NM_014873\|*LPGAT1* | 3 | N/A | 2603-[AUGUUCUCUCUCUUCUGUUU]-2622 | Cleavage |
| NM_001256272\|*VSX1* | 3 | N/A | 940-[UUUUUCACUUUCUUCUGUUG]-959 | Cleavage |
| NM_001256271\|*VSX1* | 3 | N/A | 954-[UUUUUCACUUUCUUCUGUUG]-973 | Cleavage |
| NM_001659\|*ARF3* | 3 | N/A | 1007-[AUCUUCAUUCUCUUUUGUUA]-1026 | Cleavage |
| NM_005737\|*ARL4C* | 3 | N/A | 2044-[CUGCUUUUUCUCUUUUGUCG]-2063 | Cleavage |
| NM_001018052\|*POLR3H* | 3 | N/A | 3385-[CUGAUUAGUCUCUUCUGUCA]-3404 | Cleavage |
| NM_007190\|*SEC23IP* | 3 | N/A | 1007-[AUGCUGAUUUUCUUUUGUUA]-1026 | Cleavage |
| NM_001080414\|*CCDC88C* | 3.5 | N/A | 1015-[GGAGUCACUCUCUUCUGUCG]-1034 | Cleavage |
| NM_003848\|*SUCLG2* | 3.5 | N/A | 210-[GUUCUCAUACUUUUCUGUCA]-229 | Cleavage |
| NM_024743\|*UGT2A3* | 3.5 | N/A | 185-[GUCUUUACUCUCUUCUCUCA]-204 | Cleavage |
| NM_006091\|*CORO2B* | 3.5 | N/A | 1486-[GUGUCCACUCUUUUCUUUCA]-1505 | Cleavage |
| NM_001178037\|*DPP10* | 3.5 | N/A | 268-[GUGCAUGUUUUCUUCUGUUA]-287 | Cleavage |
| NM_020868\|*DPP10* | 3.5 | N/A | 268-[GUGCAUGUUUUCUUCUGUUA]-287 | Cleavage |
| NM_007375\|*TARDBP* | 3.5 | N/A | 1116-[GUGUGUGUUCUCUUCUGUUA]-1135 | Cleavage |
| NM_033107\|*GTPBP10* | 3.5 | N/A | 1315-[UAUUUCACUUUCUUUUGUCA]-1334 | Cleavage |
| NM_024577\|*SH3TC2* | 3.5 | N/A | 14787-[GAACUCAUUCUUUUCUGUUA]-14806 | Cleavage |
| NM_001143943\|EFCAB2 | 3.5 | N/A | 1120-[UAGUUCAUUCUUUUUUGUUA]-1139 | Cleavage |
| NM_199169\|*PMEPA1* | 3.5 | N/A | 309-[AUGCUUAUUUUCUUUUGUUU]-328 | Cleavage |
| NM_001112808\|*FPGT-TNNI3K* | 3.5 | N/A | 370-[UUGUUCAUUUUCUUUUCUCA]-389 | Cleavage |
| NM_018013\|*SOBP* | 3.5 | N/A | 1073-[CUGUUUGCUUUCUCCUGUCA]-1092 | Cleavage |
| NM_031448\|*C19orf12* | 3.5 | N/A | 3041-[CUGUUCAUUUCCUUUUGUCA]-3060 | Translation |
| NM_001256046\|*C19orf12* | 3.5 | N/A | 3088-[CUGUUCAUUUCCUUUUGUCA]-3107 | Translation |
| NM_001077199\|*SREK1* | 3.5 | N/A | 2512-[UUGUUUAUUCUUUUCUUUCA]-2531 | Cleavage |
| NM_018351\|*FGD6* | 3.5 | N/A | 3694-[CUGUUUAUUUUCUUCUGUAA]-3713 | Cleavage |
| NM_006699\|*MAN1A2* | 3.5 | N/A | 2491-[UUCUUUACUUUCUUCUGUCU]-2510 | Cleavage |
| NM_001040445\|*ASB1* | 3.5 | N/A | 911-[UUGAUUGCUCUCUUUUGUCU]-930 | Cleavage |
| NM_001031848\|*SERPINB8* | 3.5 | N/A | 220-[AUGCUCUUUGUCUUUUGUCA]-239 | Translation |
| NM_001142289\|*MGRN1* | 3.5 | N/A | 811-[CUCCUCAUUCUCUUUUGCCA]-830 | Cleavage |
| NM_001142290\|*MGRN1* | 3.5 | N/A | 3420-[CUCCUCAUUCUCUUUUGCCA]-3439 | Cleavage |
| NM_001128933\|*SYNPO2* | 3.5 | N/A | 1602-[CUGCUCUUUUUCUGCUGUCA]-1621 | Cleavage |
| NM_001145440\|*TYW1B* | 3.5 | N/A | 463-[UUGAUCCCUUUCUUCUGUCU]-482 | Cleavage |
| NM_004674\|*ASH2L* | 3.5 | N/A | 2-[CAGGUCCCUCUUUUCUGUCA]-21 | Cleavage |
| NM_014048\|*MKL2* | 3.5 | N/A | 1106-[UGGCCCUCUUUCUUCUGUCA]-1125 | Cleavage |
| NM_006241\|*PPP1R2* | 3.5 | N/A | 898-[UUUCUCUCACUCUUCUGUCA]-917 | Cleavage |
| NM_033087\|*ALG2* | 4 | N/A | 432-[GUGUUCACUGUCAUCUGUUA]-451 | Translation |
| NM_014848\|*SV2B* | 4 | N/A | 1791-[AUGUUUACUCUCCUUCUGUCA]-1811 | Cleavage |
| NM_021174\|*KIAA1967* | 4 | N/A | 294-[GUGCUGACUUUCUCCUGUCC]-313 | Cleavage |
| NM_145071\|*CISH* | 4 | N/A | 691-[GUGUCCACUCUCUUCUGCCC]-710 | Cleavage |
| NM_004657\|*SDPR* | 4 | N/A | 328-[GUGAUUCAUUCUUUUUUGUCA]-348 | Cleavage |
| NM_015527\|*TBC1D10B* | 4 | N/A | 764-[GUUUACAUUCUCUUCUGUCU]-783 | Cleavage |
| NM_016101\|*NIP7* | 4 | N/A | 554-[AGGUUCAUUUUCUUCUGUUU]-573 | Cleavage |
| NM_001031681\|*CTNS* | 4 | N/A | 327-[UGGCUUACUCUCUUCUGCCC]-346 | Cleavage |
| NM_004937\|*CTNS* | 4 | N/A | 692-[UGGCUUACUCUCUUCUGCCC]-711 | Cleavage |
| NM_001112732\|*MCF2L* | 4 | N/A | 1843-[CGCUUUGCUUUCUUCUGUCA]-1862 | Cleavage |
| NM_001166271\|*SPATA13* | 4 | N/A | 485-[GCUCUUACUCUCUUCUGUAA]-504 | Cleavage |
| NM_014636\|*RALGPS1* | 4 | N/A | 2933-[AAACUCACUCUCUUUUGCCA]-2952 | Cleavage |
| NM_003162\|*STRN* | 4 | N/A | 986-[GUGCUCUUUCUUCUUUGUCA]-1005 | Cleavage |
| NM_017654\|*SAMD9* | 4 | N/A | 1759-[AAGCUCACUCUUUAUUGUUA]-1778 | Cleavage |
| NM_015962\|*FCF1* | 4 | N/A | 631-[AUGCUCACUCUUUUUUUUUU]-650 | Cleavage |
| NM_175929\|*FGF14* | 4 | N/A | 108-[AUGCUUAUUCUUUGCUGUCU]-127 | Cleavage |
| NM_001040172\|*HTR4* | 4 | N/A | 29-[UUGCUCAUUUUUUUUUCUUA]-48 | Cleavage |
| NM_024408\|*NOTCH2* | 4 | N/A | 256-[AUGUUUACUCUCUUCUAUUU]-275 | Cleavage |
| NM_005578\|*LPP* | 4 | N/A | 1848-[UGGCUCAUUUGCUUUUGUCA]-1867 | Translation |
| NM_001161429\|*RANBP3L* | 4 | N/A | 352-[AUGUUUAUUUUUUUCAGUCA]-371 | Cleavage |
| NM_005048\|*PTH2R* | 4 | N/A | 255-[UUGUUCAUUUUUUUCUGCUA]-274 | Cleavage |
| NM_145733\|*SEPT3* | 4 | N/A | 646-[GAGUUGACUUUCUUCUGUCC]-665 | Cleavage |
| NM_019106\|*SEPT3* | 4 | N/A | 2857-[GAGUUGACUUUCUUCUGUCC]-2876 | Cleavage |
| NM_002387\|*MCC* | 4 | N/A | 1644-[AUGUUCACUGGUUUCUGUCA]-1663 | Translation |
| NM_001195214\|*YIPF6* | 4 | N/A | 5111-[UUGUUUACUCUGUUCUUUCA]-5130 | Cleavage |
| NM_134262\|*RORA* | 4 | N/A | 982-[AUGCUCAUUUUUUUUUUUUA]-1001 | Cleavage |
| NM_017730\|*QRICH1* | 4 | N/A | 250-[UGGCUGACUCUUUUUUGUCU]-269 | Cleavage |
| NM_003286\|*TOP1* | 4 | N/A | 498-[GUCGUCACUCUCUAUUGUCA]-517 | Cleavage |
| NM_032012\|*TMEM245* | 4 | N/A | 4024-[GAGUUCUUUUUUUUCUGUCA]-4043 | Cleavage |
| NM_000611\|*CD59* | 4 | N/A | 1381-[GUGCCAGCUUUCUUCUGUUG]-1400 | Cleavage |
| NM_144717\|*IL20RB* | 4 | N/A | 786-[GUGCUC-CUUUUUUCUGUUG]-804 | Cleavage |
| NM_023929\|*ZBTB10* | 4 | N/A | 1073-[AUGUGCAUUCUCUUUUGUUU]-1092 | Cleavage |
| NM_001202543\|*CUX1* | 4 | N/A | 2923-[UUGUUUUCUCUUUUCUGUUU]-2942 | Cleavage |
| NM_033122\|*CABS1* | 4 | N/A | 180-[AUCUUCAUUCUUUUCUGGCA]-199 | Cleavage |
| NM_001100400\|*PDS5A* | 4 | N/A | 272-[AUGUGCAUUUUCUUCUUUCA]-291 | Cleavage |
| NM_001193536\|*DOCK8* | 4 | N/A | 753-[UUGCUUUUUUUCUUAUGUCA]-772 | Cleavage |
| NM_007021\|*C10orf10* | 4 | N/A | 1049-[AUGUUUUCUGUCUUCUGUUA]-1068 | Translation |
| NM_006418\|*OLFM4* | 4 | N/A | 656-[CUGCUUUUUCUUUUCUCUCA]-675 | Cleavage |
| NM_001277093\|*ZNF550* | 4 | N/A | 1101-[UUGUUUUUCCUCUUCUGUCA]-1120 | Cleavage |
| NM_001277092\|*ZNF550* | 4 | N/A | 1663-[UUGUUUUUCCUCUUCUGUCA]-1682 | Cleavage |
| NM_001277091\|*ZNF550* | 4 | N/A | 2010-[UUGUUUUUCCUCUUCUGUCA]-2029 | Cleavage |
| NM_199000\|*LHFPL3* | 4 | N/A | 2205-[UUUCUCAUUCUGUUCUGUUG]-2224 | Cleavage |
| NM_015233\|*MTUS2* | 4 | N/A | 2255-[UUGUUUUUUUUCUUUUGUUA]-2274 | Cleavage |
| NM_001277090\|*ZNF550* | 4 | N/A | 2596-[UUGUUUUUCCUCUUCUGUCA]-2615 | Cleavage |
| NM_006665\|*HPSE* | 4 | N/A | 1513-[UUGCUGAUUUUCUUCUUUCG]-1532 | Cleavage |
| NM_001143958\|*TMEM30A* | 4 | N/A | 1044-[AUGCACAUUCUCUUUAGUUA]-1063 | Cleavage |
| NM_001172638\|*ZFP62* | 4.5 | N/A | 1097-[GGGCUCACUCUCUCUUUGUCA]-1117 | Cleavage |
| NM_001001709\|*C9orf170* | 4.5 | N/A | 1467-[GUGUUUGUUUUCUUUUGUUC]-1486 | Cleavage |
| NM_025140\|*CCDC92* | 4.5 | N/A | 579-[GUGUUCAUAUUUUUUUGUUA]-598 | Cleavage |
| NM_006333\|*C1D* | 4.5 | N/A | 437-[AUGUUCAUUUUUUUCUGUAU]-456 | Cleavage |
| NM_001142650\|*HNRPLL* | 4.5 | N/A | 681-[GUGUUCAUUUUCAUUUGUUG]-700 | Cleavage |
| NM_001649\|*SHROOM2* | 4.5 | N/A | 408-[GUGCUCAUCUUCUCUUGUCA]-427 | Cleavage |
| NM_001031713\|*MCUR1* | 4.5 | N/A | 2500-[GUGUUCACUUUAUUUGGUCA]-2519 | Cleavage |
| NM_004731\|*SLC16A7* | 4.5 | N/A | 3325-[AUACUCAUUCUUUUCUGGCU]-3344 | Cleavage |
| NM_001119\|*ADD1* | 4.5 | N/A | 1291-[GUGCUGCCUCUCUUCUGUGU]-1310 | Cleavage |
| NM_014190\|*ADD1* | 4.5 | N/A | 1643-[GUGCUGCCUCUCUUCUGUGU]-1662 | Cleavage |
| NM_001081573\|*GAB3* | 4.5 | N/A | 2522-[UGUUUCAUUUUCUUCUGUCU]-2541 | Cleavage |
| NM_001171610\|*LDB3* | 4.5 | N/A | 1515-[CAACUCACUCUAUUCUGUCC]-1534 | Cleavage |
| NM_003221\|*TFAP2B* | 4.5 | N/A | 973-[CAUUUCAUUCUCUUCUGUUU]-992 | Cleavage |
| NM_138991\|*BACE2* | 4.5 | N/A | 322-[UGGCUCGUUCUCUUCUCUCU]-341 | Cleavage |
| NM_138992\|*BACE2* | 4.5 | N/A | 519-[UGGCUCGUUCUCUUCUCUCU]-538 | Cleavage |
| NM_001134831\|*AHI1* | 4.5 | N/A | 1592-[AAGCUUGUUUUUUUCUGUCU]-1611 | Cleavage |
| NM_015423\|*AASDHPPT* | 4.5 | N/A | 980-[UUACUCACUUUUUUCUGUGU]-999 | Cleavage |
| NM_001002862\|*DERL3* | 4.5 | N/A | 1544-[GUGCCCAUUCUCUUCAGUAA]-1563 | Cleavage |
| NM_198440\|*DERL3* | 4.5 | N/A | 1735-[GUGCCCAUUCUCUUCAGUAA]-1754 | Cleavage |
| NM_001165947\|*HTR2A* | 4.5 | N/A | 124-[UGCCUCAUUUUAUUCUGUCA]-143 | Cleavage |
| NM_006769\|*LMO4* | 4.5 | N/A | 809-[CUGCUUUUUCUCUUCUGUAU]-828 | Cleavage |
| NM_014906\|*PPM1E* | 4.5 | N/A | 1810-[GUCCUCACUCAUAUCUGUCA]-1829 | Translation |
| NM_001127176\|*MRO* | 4.5 | N/A | 2587-[ACCUUCGUUUUCUUCUGUUA]-2606 | Cleavage |
| NM_001695\|*ATP6V1C1* | 4.5 | N/A | 3698-[GUGAUUAUCUUCUUCUGUUA]-3717 | Cleavage |
| NM_199454\|*PRDM16* | 4.5 | N/A | 3895-[UGUUUUGUUCUUUUCUGUCA]-3914 | Cleavage |
| NM_018718\|*CEP41* | 4.5 | N/A | 4222-[AAGCUCGUUAUCUUCUGUCC]-4241 | Translation |
| NM_004096\|*EIF4EBP2* | 4.5 | N/A | 1627-[AGCCUCAUUCUUUUCUGUGA]-1646 | Cleavage |
| NM_001079526\|*IKZF2* | 4.5 | N/A | 6753-[GUGCUAAUUUUCUUUUUUUA]-6772 | Cleavage |
| NM_001103184\|*FMN1* | 4.5 | N/A | 7414-[UGUUUCACUUUCUUCUUUCA]-7433 | Cleavage |
| NM_024692\|*CLIP4* | 4.5 | N/A | 295-[UUGCUUAUUCUGUUUUGUUU]-314 | Cleavage |
| NM_203371\|*FIBIN* | 4.5 | N/A | 1481-[AUGUUCAUAUUCUUCUGUUC]-1500 | Cleavage |
| NM_017666\|*ZNF280C* | 4.5 | N/A | 304-[CUGUUCAUCUUUUUCUGUCU]-323 | Cleavage |
| NM_018667\|*SMPD3* | 4.5 | N/A | 2525-[AUGUUCAUUUUCAUUUGUCC]-2544 | Cleavage |
| NM_015575\|*GIGYF2* | 4.5 | N/A | 338-[CAGCUUGCUUUCUCUUGUCA]-357 | Cleavage |
| NM_018958\|*NPAP1* | 4.5 | N/A | 164-[GUGCUCCCUUUCU-CUGUCA]-182 | Cleavage |
| NM_022648\|*TNS1* | 4.5 | N/A | 2841-[AUGUUUAUCUUCUUCUGUCU]-2860 | Cleavage |
| NM_005195\|*CEBPD* | 4.5 | N/A | 336-[AUGCUCACUUUUUUAUAUUA]-355 | Cleavage |
| NM_006571\|*DCTN6* | 4.5 | N/A | 100-[AUGUUCACUUUAUUUUGUAA]-119 | Cleavage |
| NM_001037666\|*GATSL3* | 4.5 | N/A | 450-[CAUCUCUCUCUCUUCUGUUU]-469 | Cleavage |
| NM_001190794\|*NCF2* | 4.5 | N/A | 537-[UUGUUUGUUUUCUUUUUUCA]-556 | Cleavage |
| NM_014977\|*ACIN1* | 4.5 | N/A | 429-[GUUCCCAGUCUCUUCUGUCC]-448 | Cleavage |
| NM_014892\|*SCAF8* | 4.5 | N/A | 79-[UUGUUCACUUUUGUCUGCCA]-98 | Cleavage |
| NM_001193380\|*IL17RE* | 4.5 | N/A | 239-[CUGCUCGCUUACUUCAGUCG]-258 | Translation |
| NM_018713\|*SLC30A10* | 4.5 | N/A | 638-[AUGUUUACUUUCUGCUGUGA]-657 | Cleavage |
| NM_017798\|*YTHDF1* | 4.5 | N/A | 454-[UUGUUCAUUCUGAUUUGUCA]-473 | Cleavage |
| NM_003934\|*FUBP3* | 4.5 | N/A | 888-[UUGUUUGCUUUUUUCUUUUA]-907 | Cleavage |
| NM_004961\|*GABRE* | 4.5 | N/A | 1285-[ACCAUGAUUCUCUUCUGUCA]-1304 | Cleavage |
| NM_004236\|*COPS2* | 4.5 | N/A | 1557-[AUGCUUAUUCGGUUUUGUCA]-1576 | Translation |
| NM_004236\|*COPS2* | 4.5 | N/A | 2222-[GCGUUCUUUUUUUUCUGUUA]-2241 | Cleavage |
| NM_017944\|*USP47* | 4.5 | N/A | 1331-[UUGUUCAUUCUGUUGUGUUA]-1350 | Cleavage |
| NM_020940\|*FAM160B1* | 4.5 | N/A | 1427-[CUGCUCAUUUUGUUGUGUUA]-1446 | Cleavage |
| NM_001145345\|*ZNF566* | 4.5 | N/A | 2313-[UUGUUUACUUUCUUUUCUUG]-2332 | Cleavage |
| NM_152795\|*HIF3A* | 5 | N/A | 2763-[GUUUUUCCUUUUUUUUGUUA]-2782 | Cleavage |
| NM_033121\|*ANKRD13A* | 5 | N/A | 1512-[UUGUUU-UUUUUUUUUGUCA]-1530 | Cleavage |
| NM_004505\|*USP6* | 5 | N/A | 919-[GUGGUUAUUCUUUUCUGUGU]-938 | Cleavage |
| NM_080817\|*GPR82* | 5 | N/A | 982-[GUGCAUAUUUUCUUCUGUAU]-1001 | Cleavage |
| NM_024408\|*NOTCH2* | 5 | N/A | 2729-[UUGCUUCCUCUGUUUUGUUU]-2748 | Cleavage |
| NM_006361\|*HOXB13* | 5 | N/A | 1016-[GUGUUCAUUCUCUGAUGUCC]-1035 | Cleavage |
| NM_025205\|*MED28* | 5 | N/A | 654-[GUGUUUACUUUUUUUUUUUG]-673 | Cleavage |
| NM_014398\|*LAMP3* | 5 | N/A | 1595-[UUGUUCACUUUAUUCUGUAG]-1614 | Cleavage |
| NM_001040202\|*PAQR3* | 5 | N/A | 2508-[GUGCUUGUCUUUUUUUGUUA]-2527 | Cleavage |
| NM_178815\|*ARL5B* | 5 | N/A | 2015-[UGGCUCAAUUUUCUUCUGUCA]-2035 | Cleavage |
| NM_212472\|*PRKAR1A* | 5 | N/A | 1967-[CUGUUUACUCCCUUCUGUAG]-1986 | Translation |
| NM_174911\|*FAM84B* | 5 | N/A | 2021-[GUGUUUAUUCUCUUUGCUCA]-2040 | Cleavage |
| NM_007011\|*ABHD2* | 5 | N/A | 6101-[GUGUUUACUCUUUUCAUUCG]-6120 | Cleavage |
| NM_007011\|*ABHD2* | 5 | N/A | 5854-[UCUUUUACACUCUUCUGUUA]-5873 | Cleavage |
| NM_017759\|*INO80D* | 5 | N/A | 4795-[GCCCUUAUUUUUUUUUGUUA]-4814 | Cleavage |
| NM_002867\|*RAB3B* | 5 | N/A | 8913-[CUGUUUACUUCUCUUCUGUCU]-8933 | Translation |
| NM_001779\|*CD58* | 5 | N/A | 197-[GUGCUUGCU-UUUUUUGUCA]-215 | Translation |
| NM_001193331\|*C9orf3* | 5 | N/A | 1377-[CUGUUCAGCUCUCUCCUGUCA]-1397 | Cleavage |
| NM_024900\|*PHF17* | 5 | N/A | 1680-[GUACUAACUCUUUUCUGUAC]-1699 | Cleavage |
| NM_001199140\|*AMMECR1L* | 5 | N/A | 1557-[UUUUGCAUUUUCUUCUGUCA]-1576 | Cleavage |
| NM_004999\|*MYO6* | 5 | N/A | 2621-[AUGCUCAUUCUGCUUCUGUAA]-2641 | Cleavage |
| NM_182715\|*SYPL1* | 5 | N/A | 529-[GUGUAUAUUUUUUUCUGUUU]-548 | Cleavage |
| NM_001003937\|*TSPYL6* | 5 | N/A | 916-[GUGUACACUUUCUUUUCUCU]-935 | Cleavage |
| NM_004884\|*IGDCC3* | 5 | N/A | 1255-[UUUGUUACUCUUUUCUGUUG]-1274 | Cleavage |
| NM_080874\|*ASB5* | 5 | N/A | 1343-[GGGCUUAUUUUCUUCUGACU]-1362 | Cleavage |
| NM_014824\|*FCHSD2* | 5 | N/A | 1228-[GUGCUGACUGUCUGCUGUCC]-1247 | Translation |
| NM_015302\|*HAUS5* | 5 | N/A | 1093-[GUUCUUGUUUUCUUUUGUUU]-1112 | Cleavage |
| NM_001146686\|*GMNC* | 5 | N/A | 1477-[GUGCUAGUUUUCUUCUUUCC]-1496 | Cleavage |
| NM_173529\|*C18orf54* | 5 | N/A | 2594-[UGUUUCAUUUUUUUCUGUCC]-2613 | Cleavage |
| **TAPIR** | | | | |
| **Target accession** | **Score** | **MFE ratio** | **mRNA target aligned fragment (5’-3’)** | |
| **NM_016946\|*F11R*** | **3** | **0.8** | **504-[GGGCCCACUCUCUUCUGUCU]-523** | |
| NM_001178097\|*C12orf74* | 4 | 0.76 | 190-[UCGCUCACUUUCUUCUGUCC]-209 | |
| NM_001080414\|*CCDC88C* | 3.5 | 0.77 | 1015-[GGAGUCACUCUCUUCUGUCG]-1034 | |
| NM_001172638\|*ZFP62* | 4 | 0.78 | 1097-[GGGCUCACUCUCUCUUUGUCA]-1117 | |

The cDNA library “*Homo* *sapiens* (human), transcript, Human genomic sequencing project” (available at psRNATarget server) was aligned with miR156e mature sequence (5'-UGACAGAAGAGAGUGAGCAC-3'). Outputs highlighted in bold remarks common putative target genes to the three algorithms. N/A: not applicable.

**Table S3. Bioinformatic analysis to predict putative human target genes of plant miR159 performed by psRNATarget (scoring schemas V1 and V2) and TAPIR software.**

| **Plant miR159 putative target genes** | | | | |
| --- | --- | --- | --- | --- |
| **psRNATarget. Scoring Schema V1** | | | | |
| **Target accession** | **Expectation** | **UPE** | **mRNA target aligned fragment (5’-3’)** | **Inhibitory effect** |
| NM_133265\|*AMOT* | 2.5 | 10.984 | 2221-[GAGAGCUACUUUCUAUCCAAA]-2241 | Cleavage |
| **NM_005444\|*RQCD1*** | **3.0** | **24.075** | **909-[UGGACCUCACCUUCAAUCCAAG]-930** | **Cleavage** |
| NM_014788\|*TRIM14* | 3.0 | 16.076 | 1348-[AAGAGCACCCUUAGAUCCAGA]-1368 | Translation |
| NM_001204456\|*RBAK* | 3.0 | 10.411 | 119-[UAGAG-UCAUUUUAAUCCAAA]-138 | Cleavage |
| **psRNATarget. Scoring Schema V2** | | | | |
| **Target accession** | **Expectation** | **UPE** | **mRNA target aligned fragment (5’-3’)** | **Inhibitory effect** |
| NM_014906\|*PPM1E* | 2.5 | N/A | 2442-[AGUAGUUCCCUUCAGUUCAAA]-2462 | Cleavage |
| NM_001024455\|*RGAG4* | 2.5 | N/A | 1101-[CUGAGCUCCCUUCAAUUUCAA]-1121 | Cleavage |
| NM_133265\|*AMOT* | 3.0 | N/A | 2221-[GAGAGCUACUUUCUAUCCAAA]-2241 | Cleavage |
| NM_000476\|*AK1* | 3.0 | N/A | 1368-[CUGGGCUCCCUUUAAGCCAAG]-1388 | Cleavage |
| NM_005578\|*LPP* | 3.0 | N/A | 5396-[AGAAUUUCCCUUCAAUCCAGA]-5416 | Cleavage |
| NM_005544\|*IRS1* | 3.0 | N/A | 943-[GGGUGAUUCCUUCAAUUCAAA]-963 | Cleavage |
| NM_145010\|*ENKUR* | 3.5 | N/A | 469-[UAGAGCUGUUUUCAACCCAAA]-489 | Cleavage |
| NM_001001852\|*PIM3* | 3.5 | N/A | 861-[UGGGGGUUCUUUCAGUUCAAA]-881 | Cleavage |
| NM_014788\|*TRIM14* | 3.5 | N/A | 1348-[AAGAGCACCCUUAGAUCCAGA]-1368 | Cleavage |
| NM_024557\|*RIC3* | 3.5 | N/A | 3438-[CAAAGUUCUCUUCAAUCUGGA]-3458 | Cleavage |
| NM_031216\|*SEH1L* | 3.5 | N/A | 778-[CUUAGCUUCCUUCAAAUCAAA]-798 | Cleavage |
| NM_001198595\|*STON1* | 3.5 | N/A | 2989-[GUUAGUUCCCUUCAUUCCAGA]-3009 | Cleavage |
| NM_018010\|*IFT57* | 4.0 | N/A | 1133-[UAGACCUCUCUUUAGUUUAAG]-1153 | Cleavage |
| NM_178509\|*STXBP4* | 4.0 | N/A | 3266-[UAGGACUUUUUUUAGUCCAAA]-3286 | Cleavage |
| NM_015074\|*KIF1B* | 4.0 | N/A | 1395-[UACAGCUCCCUUUGAUCAAAG]-1415 | Cleavage |
| NM_012152\|*LPAR3* | 4.0 | N/A | 247-[CAGAGCUCCUGUCAGUCCAGC]-267 | Translation |
| NM_000325\|*PITX2* | 4.0 | N/A | 152-[GAGAGCUCCUUUGAUUUCAAA]-172 | Cleavage |
| NM_001146702\|*KDM5C* | 4.0 | N/A | 326-[CAGGGCUCCCUGUGAUCUGAA]-346 | Translation |
| NM_004187\|*KDM5C* | 4.0 | N/A | 549-[CAGGGCUCCCUGUGAUCUGAA]-569 | Translation |
| NM_014677\|*RIMS2* | 4.0 | N/A | 1183-[AAGAGCUUUCUUUAUUUCAAG]-1203 | Cleavage |
| NM_001166270\|*HAUS4* | 4.0 | N/A | 79-[UGGGGCUGCCUUCAGUACAAG]-99 | Cleavage |
| NM_001204106\|*BCL2L11* | 4.0 | N/A | 1721-[UAGAGAUGAUUUCAAUCCAAA]-1741 | Cleavage |
| NM_001204111\|*BCL2L11* | 4.0 | N/A | 1806-[UAGAGAUGAUUUCAAUCCAAA]-1826 | Cleavage |
| NM_001204112\|*BCL2L11* | 4.0 | N/A | 1916-[UAGAGAUGAUUUCAAUCCAAA]-1936 | Cleavage |
| NM_001204108\|*BCL2L11* | 4.0 | N/A | 1931-[UAGAGAUGAUUUCAAUCCAAA]-1951 | Cleavage |
| NM_138623\|*BCL2L11* | 4.0 | N/A | 1933-[UAGAGAUGAUUUCAAUCCAAA]-1953 | Cleavage |
| NM_138625\|*BCL2L11* | 4.0 | N/A | 1936-[UAGAGAUGAUUUCAAUCCAAA]-1956 | Cleavage |
| NM_138624\|*BCL2L11* | 4.0 | N/A | 1943-[UAGAGAUGAUUUCAAUCCAAA]-1963 | Cleavage |
| NM_001204107\|*BCL2L11* | 4.0 | N/A | 1987-[UAGAGAUGAUUUCAAUCCAAA]-2007 | Cleavage |
| NM_001522\|*GUCY2F* | 4.0 | N/A | 48-[GAAAGCUCACUUCAGUUCAAG]-68 | Cleavage |
| NM_001553\|*IGFBP7* | 4.0 | N/A | 71-[AUAAGUUUCUUUUAAUCCAAU]-91 | Cleavage |
| NM_018843\|*SLC25A40* | 4.0 | N/A | 488-[GCCUGUUUCCUUUAAUUCAAA]-508 | Cleavage |
| NM_030773\|*TUBB1* | 4.0 | N/A | 263-[AAGUGCUCCCUUUGUUUCAAA]-283 | Cleavage |
| NM_001040424\|*PRDM15* | 4.0 | N/A | 942-[CAGGAUUCUCUGCAAUCCAAA]-962 | Translation |
| NM_015085\|*RAP1GAP2* | 4.0 | N/A | 1478-[AAGAGCCCCUUUCAGUGCAGA]-1498 | Cleavage |
| NM_001080383\|*GJC1* | 4.0 | N/A | 2105-[CACAGUUUCCUUCAAAUCAAA]-2125 | Cleavage |
| NM_001080383\|*GJC1* | 4.0 | N/A | 4150-[CACAGUUUCCUUCAAAUCAAA]-4170 | Cleavage |
| NM_020774\|*MIB1* | 4.0 | N/A | 3340-[GGUUGCUUUUUUUAAUCCAAA]-3360 | Cleavage |
| NM_012405\|*ICMT* | 4.0 | N/A | 798-[AUCAGUUUCUUUUGAUCCAAG]-818 | Cleavage |
| NM_003559\|*PIP4K2B* | 4.0 | N/A | 432-[CUCAGUUCCCUUCAAUUAAAG]-452 | Cleavage |
| NM_000990\|*RPL27A* | 4.0 | N/A | 1054-[UUGAGCUUUUUUUGACCCAAA]-1074 | Cleavage |
| NM_182503\|*ADAT2* | 4.0 | N/A | 3234-[UUUAGCUUCUUUUAGUCUAGA]-3254 | Cleavage |
| NM_001146213\|*TBC1D15* | 4.0 | N/A | 3614-[CUGAGCUCCCAUUAAUUAAAA]-3634 | Translation |
| NM_001198846\|*RBM14*-*RBM4* | 4.0 | N/A | 338-[AGGGUCUCUUUUUGGUCCAAA]-358 | Cleavage |
| NM_001198845\|*RBM14*-*RBM4* | 4.0 | N/A | 366-[AGGGUCUCUUUUUGGUCCAAA]-386 | Cleavage |
| NM_014608\|*CYFIP1* | 4.0 | N/A | 106-[GGGGGUGCUUUUCGAUCUAAA]-126 | Cleavage |
| NM_153267\|*MAMDC2* | 4.0 | N/A | 35-[CAUACCUCUCUUCAAUCAAAA]-55 | Cleavage |
| NM_001114107\|*PDLIM3* | 4.0 | N/A | 778-[CAGAGACUCUUUCAAUUUAAA]-798 | Cleavage |
| NM_152556\|*C7orf60* | 4.0 | N/A | 11-[AAAAGCCCCUUUCAGUCCAGG]-31 | Cleavage |
| NM_152900\|*MAGI3* | 4.0 | N/A | 1394-[GAGAAAUCUCUUCCAUCCAAA]-1414 | Cleavage |
| NM_005370\|*RAB8A* | 4.0 | N/A | 1168-[UUAAGCUGCUGUCAAUCCAAA]-1188 | Translation |
| NM_004686\|*MTMR7* | 4.0 | N/A | 2448-[CUGAGCUGUCCUUCAAUUUAAG]-2469 | Cleavage |
| **NM_005444\|*RQCD1*** | **4.5** | **N/A** | **909-[UGGACCUCACCUUCAAUCCAAG]-930** | **Cleavage** |
| NM_004705\|*PRKRIR* | 4.5 | N/A | 866-[UAUAGUUUAUUUCAAUCUAAA]-886 | Cleavage |
| NM_001170570\|*CXorf56* | 4.5 | N/A | 846-[UAAAGCUUUCUUUACUUCAAA]-866 | Cleavage |
| NM_181742\|*ORC4* | 4.5 | N/A | 295-[AAGAGCUCACUUCAACCUAAC]-315 | Cleavage |
| NM_003853\|*IL18RAP* | 4.5 | N/A | 10-[UGGAGCCCCCUCCAGUCCAGU]-30 | Translation |
| NM_001242903\|*AKAP1* | 4.5 | N/A | 395-[GAGGGUUUCUUUUACUUCAAA]-415 | Cleavage |
| NM_003488\|*AKAP1* | 4.5 | N/A | 395-[GAGGGUUUCUUUUACUUCAAA]-415 | Cleavage |
| NM_001142699\|*DLG2* | 4.5 | N/A | 2655-[CAGAGCUCUCUCCAACCUGAA]-2675 | Translation |
| NM_024776\|*PEAK1* | 4.5 | N/A | 1739-[CAGAGUUUCCUUAAAGCCAAG]-1759 | Cleavage |
| NM_001113239\|*HIPK2* | 4.5 | N/A | 6844-[GAGAGUUUCUUUUGAUUCAUA]-6864 | Cleavage |
| NM_001098790\|*MID1IP1* | 4.5 | N/A | 882-[UAAAACUUUUUUUAAUCCAGA]-902 | Cleavage |
| NM_024120\|*NDUFAF5* | 4.5 | N/A | 1115-[UAUUGUUCCCUUCAGUUUGAA]-1135 | Cleavage |
| NM_000542\|*SFTPB* | 4.5 | N/A | 427-[UACAGCUACUUGCAAUUCAAA]-447 | Translation |
| NM_000138\|*FBN1* | 4.5 | N/A | 2320-[UGGAACUCUUUUUAAUCCUGA]-2340 | Cleavage |
| NM_001987\|*ETV6* | 4.5 | N/A | 1533-[UACAAUUUCUUUUAAUCCAGA]-1553 | Cleavage |
| NM_017520\|*MPHOSPH8* | 4.5 | N/A | 20-[CGGAGUUCUCUUCAGACCGAU]-40 | Cleavage |
| NM_006805\|*HNRNPA0* | 4.5 | N/A | 1617-[CAGAGUACCUUUUAAUCUAGU]-1637 | Cleavage |
| NM_003861\|*DCAF5* | 4.5 | N/A | 133-[UGCUGCUCCCUUCUAUCCAAC]-153 | Cleavage |
| NM_001204456\|*RBAK* | 4.5 | N/A | 119-[UAGAG-UCAUUUUAAUCCAAA]-138 | Cleavage |
| NM_021964\|*ZNF148* | 4.5 | N/A | 3430-[UGUUGCUUUCUUUAAUCCAAU]-3450 | Cleavage |
| NM_001160102\|*LPO* | 4.5 | N/A | 18-[GAAAGUUCCCUUUGGUCCACA]-38 | Cleavage |
| NM_014326\|*DAPK2* | 4.5 | N/A | 43-[CGGGGCUCCCUUCUGUGCAGA]-63 | Cleavage |
| NM_021190\|*PTBP2* | 4.5 | N/A | 1177-[AACAGUUCUUGUCAAUCCGAA]-1197 | Translation |
| NM_194456\|*KRIT1* | 4.5 | N/A | 1537-[UUCAGUUCCCUUCAUUCUAAU]-1557 | Cleavage |
| NM_134325\|*SLC26A9* | 4.5 | N/A | 1359-[CAGAGUCCCCUUUGAACCAAG]-1379 | Cleavage |
| NM_020761\|*RPTOR* | 4.5 | N/A | 794-[CGGGGCUCCUUUCCCUCCGAA]-814 | Cleavage |
| NM_003042\|*SLC6A1* | 4.5 | N/A | 401-[AGGAGUUCCUUUCCCUCCGAA]-421 | Cleavage |
| NM_052934\|*SLC26A9* | 4.5 | N/A | 1824-[CAGAGUCCCCUUUGAACCAAG]-1844 | Cleavage |
| NM_153705\|*KDELC2* | 4.5 | N/A | 1432-[AAGAGCUAUCCUCAUUCCAAA]-1452 | Translation |
| NM_001195683\|*TGFBR3* | 4.5 | N/A | 2821-[CAGAGAUUUCUUCUGUCUAAA]-2841 | Cleavage |
| NM_015564\|*LRRTM2* | 4.5 | N/A | 917-[ACAUGCUCCCUCCAAUUUAAA]-937 | Translation |
| NM_015253\|*WSCD1* | 4.5 | N/A | 1419-[GAGACCUCCCUUCAGCCUGAG]-1439 | Cleavage |
| NM_182646\|*CPEB2* | 4.5 | N/A | 814-[AAAAGCUCUCUUGAAUCUAGG]-834 | Cleavage |
| NM_201431\|*RASSF6* | 4.5 | N/A | 644-[AAGACCUUUCAUCAGUUCAAA]-664 | Translation |
| NM_001683\|*ATP2B2* | 4.5 | N/A | 3119-[CGGAGCUUUCUUUAACUCAGG]-3139 | Cleavage |
| NM_017686\|*GDAP2* | 4.5 | N/A | 6084-[ACAGGCUCUUUUCAGUUCAAC]-6104 | Cleavage |
| NM_000949\|*PRLR* | 4.5 | N/A | 3695-[CAGAGGUCCCUGCAUUCCAAG]-3715 | Translation |
| NM_001008493\|*ENAH* | 4.5 | N/A | 5835-[AACUGUUCCCUUCAAUUCCAA]-5855 | Cleavage |
| NM_021013\|*KRT34* | 4.5 | N/A | 238-[UUCAGCUCCCUGUAAUCUGAG]-258 | Translation |
| NM_198541\|*IGFL1* | 4.5 | N/A | 348-[UCAAGUUCUCUUCUAUCCAGG]-368 | Cleavage |
| NM_013393\|*FTSJ2* | 4.5 | N/A | 30-[AUUAGCUCCUUUUAAGCUAGA]-50 | Cleavage |
| NM_016619\|*PLAC8* | 4.5 | N/A | 888-[CCCAGCUCUCUUUCAUCUAAG]-908 | Cleavage |
| NM_002271\|*IPO5* | 4.5 | N/A | 1633-[GUAGGCUUUUUUUAAUUUAAA]-1653 | Cleavage |
| NM_004384\|*CSNK1G3* | 4.5 | N/A | 2569-[CUAAGCUUUCUUUAAUAUAAA]-2589 | Cleavage |
| NM_001143827\|*MAPRE2* | 4.5 | N/A | 818-[AUCAGUUUUCUGCAGUCCAAA]-838 | Translation |
| NM_001008701\|*LPHN1* | 4.5 | N/A | 1108-[AGCAGCUUUUUACAAUCCAGA]-1128 | Translation |
| NM_001128164\|*ATXN1* | 4.8 | N/A | 5470-[CUGAGCUCCCUUCUUAGUCUAAA]-5492 | Cleavage |
| NM_014906\|*PPM1E* | 5.0 | N/A | 646-[GUGAACUUUUUUUAAUUAAAA]-666 | Cleavage |
| NM_138357\|*MCU* | 5.0 | N/A | 1108-[UAGGGCUUCCUUCAAGCCCAC]-1128 | Cleavage |
| NM_152999\|*STEAP2* | 5.0 | N/A | 139-[UAGAGUUUUCUUCAAGUUAAU]-159 | Cleavage |
| NM_032854\|*CORO6* | 5.0 | N/A | 886-[UAGAGCUCUCUCCACUCUGAG]-906 | Translation |
| NM_001080427\|*THSD7B* | 5.0 | N/A | 456-[UAGAGUUUACUUUGGUUUAAA]-476 | Cleavage |
| NM_015833\|*ADARB1* | 5.0 | N/A | 315-[UAGGGCUUCCUUAAGUUUAGG]-335 | Cleavage |
| NM_021191\|*NEUROD4* | 5.0 | N/A | 2279-[UAGAGUCUUUUCUUCAGUCUAAA]-2301 | Cleavage |
| NM_025181\|*SLC35F5* | 5.0 | N/A | 742-[UAAAGCUCUGUUUAAUUUAGA]-762 | Cleavage |
| NM_003496\|*TRRAP* | 5.0 | N/A | 348-[AACAGCUCCCUUCCAUCCAUU]-368 | Cleavage |
| NM_001134659\|*PRR23A* | 5.0 | N/A | 435-[AUCCGUUCUCUCCAAUCCAGA]-455 | Translation |
| NM_178448\|*SAPCD2* | 5.0 | N/A | 230-[UGGGGCUCUCUUGAGUCCGCA]-250 | Cleavage |
| NM_005226\|*S1PR3* | 5.0 | N/A | 2582-[UAGGUCUCUUUUCAAUUAGAA]-2602 | Cleavage |
| NM_001257137\|*ITCH* | 5.0 | N/A | 1057-[UAGAGUGCUUUCUAGUCCAAA]-1077 | Translation |
| NM_001164747\|*RASSF8* | 5.0 | N/A | 3591-[AGGAGCUCUUUUCAAUGCAUU]-3611 | Cleavage |
| NM_005188\|*CBL* | 5.0 | N/A | 4568-[UAGGGAUUCUUUGGAUCCAAG]-4588 | Cleavage |
| NM_001146218\|*WRB* | 5.0 | N/A | 378-[UAGUGCUGUUUUUAAUCCAGU]-398 | Cleavage |
| NM_012458\|*TIMM13* | 5.0 | N/A | 984-[GAGGGCUCCCUGCAGGCCAGG]-1004 | Translation |
| NM_032294\|*CAMKK1* | 5.0 | N/A | 476-[CAGAGCUCUUGUCUAUUCAGA]-496 | Translation |
| NM_014737\|*RASSF2* | 5.0 | N/A | 2073-[AAGAGUUCUCUUUGAUUUGCA]-2093 | Cleavage |
| NM_170774\|*RASSF2* | 5.0 | N/A | 2073-[AAGAGUUCUCUUUGAUUUGCA]-2093 | Cleavage |
| NM_001243765\|*RABGAP1L* | 5.0 | N/A | 1973-[CAGAGCUUGUUUCAAUUUACA]-1993 | Cleavage |
| NM_018666\|*SAGE1* | 5.0 | N/A | 65-[UGAAGCUCCCUAUAAUCCUGA]-85 | Translation |
| NM_001199979\|*PLSCR2* | 5.0 | N/A | 142-[UGGAACUCAUUUAAAUUCAAA]-162 | Cleavage |
| NM_001005849\|*SUMO2* | 5.0 | N/A | 552-[UAGUUUUCCCUUCAAGUCAAG]-572 | Cleavage |
| NM_182977\|*NNT* | 5.0 | N/A | 724-[UGGAGAUCUCUUUAAUUUCGA]-744 | Cleavage |
| NM_001199922\|*SIAE* | 5.0 | N/A | 665-[AUUCUGUUCCUUCAAUCUAAA]-685 | Cleavage |
| NM_001199399\|*NEK1* | 5.0 | N/A | 998-[UAGAGGUGUCUACAGUCCAGA]-1018 | Translation |
| NM_017554\|*PARP14* | 5.0 | N/A | 2171-[UAUAGUUAUCCUCAAUCUAAA]-2191 | Translation |
| NM_014829\|*DDX46* | 5.0 | N/A | 2158-[UGGUGCUCCCUUCA-UCCAAC]-2177 | Cleavage |
| NM_001080519\|*BAHCC1* | 5.0 | N/A | 2363-[UGGAUUUUUUUUCAAUGUAAA]-2383 | Cleavage |
| NM_001263\|*CDS1* | 5.0 | N/A | 385-[UGGAGCUGCUUUUAAUUUUAG]-405 | Cleavage |
| NM_024721\|*ZFHX4* | 5.0 | N/A | 258-[UGGAACUUGUUUCAAGCCAAA]-278 | Cleavage |
| NM_016018\|*PHF20L1* | 5.0 | N/A | 26-[UGUGGCUUUCUUCAGUCAAAG]-46 | Cleavage |
| NM_001170637\|*SRGAP2* | 5.0 | N/A | 2016-[UUACAUUCCUUUCAAUCCAAC]-2036 | Cleavage |
| NM_022121\|*PERP* | 5.0 | N/A | 1608-[UGGGGUCCCCUUCCAUUCAGG]-1628 | Cleavage |
| **TAPIR** | | | | |
| **Target accession** | **Score** | **MFE ratio** | **mRNA target aligned fragment (5’-3’)** | |
| **NM_005444\|*RQCD1*** | **3.0** | **0.78** | **909-[UGGACCUCACCUUCAAUCCAAG]-930** | |

The cDNA library “*Homo sapiens* (human), transcript, Human genomic sequencing project” (available at psRNATarget server) was aligned with miR159 mature sequence (5'-UUUGGAUUGAAGGGAGCUCUA-3'). Outputs highlighted in bold remarks common putative target genes to the three algorithms. N/A: not applicable.

**Table S4. Bioinformatic analysis to predict putative human target genes of plant miR162 performed by psRNATarget (scoring schemas V1 and V2) and TAPIR software.**

| **Plant miR162 putative target genes** | | | | |
| --- | --- | --- | --- | --- |
| **psRNATarget. Scoring Schema V2** | | | | |
| **Target accession** | **Expectation** | **UPE** | **mRNA target aligned fragment (5’-3’)** | **Inhibitory effect** |
| NM_001315\|*MAPK14* | 3.5 | N/A | 827-[UUUGGUGCAGAGGUUUCUUGA]-847 | Cleavage |
| NM_139014\|*MAPK14* | 3.5 | N/A | 907-[UUUGGUGCAGAGGUUUCUUGA]-927 | Cleavage |
| NM_001136498\|*CISD3* | 3.5 | N/A | 1410-[GAUGGUGCAGAGCUUUAUUGA]-1430 | Cleavage |
| NM_004283\|*RAB3D* | 3.5 | N/A | 2831-[AGGGAAACGGAGGUUUGUUGA]-2851 | Cleavage |
| NM_001168338\|*PLG* | 4 | N/A | 465-[UUGGAUAUGGAGGUUUCUUGA]-485 | Cleavage |
| NM_024841\|*PRR5L* | 4 | N/A | 427-[AGUGAUGUGGAGGUUUAUGGG]-447 | Cleavage |
| NM_001160169\|*PRR5L* | 4 | N/A | 789-[AGUGAUGUGGAGGUUUAUGGG]-809 | Cleavage |
| NM_001005361\|*DNM2* | 4 | N/A | 407-[UGGGGUGCAGGGGUAUAUCAA]-427 | Cleavage |
| NM_001204456\|*RBAK* | 4 | N/A | 2368-[GAGUAAGCAAAGGUUUAUUGA]-2388 | Cleavage |
| NM_001720\|*BMP8B* | 4.5 | N/A | 1219-[UUGGGUGGAGAGGUUUGUCUC]-1239 | Cleavage |
| NM_001048249\|*SMIM15* | 4.5 | N/A | 176-[GUGGGUGUAGAAGUUUAUAGG]-196 | Translation |
| NM_175575\|*WFIKKN2* | 4.5 | N/A | 650-[UUGGCUGCACUGGUUUGUCGA]-670 | Translation |
| NM_002665\|*PLGLB2* | 4.5 | N/A | 584-[UUGGGUAUGGAGGUUUCUUGA]-604 | Cleavage |
| NM_018061\|*PRPF38B* | 4.5 | N/A | 608-[UCUGAUGCAGAGGUUUUUAGG]-628 | Cleavage |
| NM_201648\|*GLYAT* | 4.5 | N/A | 798-[AGGGAUGUGAUGGUUUAUUGA]-818 | Translation |
| NM_004184\|*WARS* | 4.5 | N/A | 448-[UGAUGUGGGGAGGUUUAUUGA]-468 | Cleavage |
| NM_021101\|*CLDN1* | 4.5 | N/A | 409-[AUUGAUGAAGAUGUUUAUUGG]-429 | Translation |
| NM_152750\|*CDHR3* | 4.5 | N/A | 2832-[AUGAAAGCAGGGAUUUAUUGA]-2852 | Cleavage |
| NM_004866\|*SCAMP1* | 4.5 | N/A | 656-[UAGGAUUCAGGGGUUAAUGGA]-676 | Cleavage |
| NM_014747\|RIMS3 | 5 | N/A | 2727-[AUGGAUGCAGGGGCUUGUCUG]-2747 | Cleavage |
| NM_181809\|*BMP8A* | 5 | N/A | 1441-[UUGGGUGGGGAGGUUUGUCUC]-1461 | Cleavage |
| NM_030919\|*FAM83D* | 5 | N/A | 210-[AUGGAUACAGGGGUUUGUUUU]-230 | Cleavage |
| NM_138570\|*SLC38A10* | 5 | N/A | 117-[CCAGAUGUGGAGGUUUAUUUU]-137 | Cleavage |
| NM_145185\|*MAP2K7* | 5 | N/A | 1039-[GUGGAUGUGGGGGUUUGAGGA]-1059 | Cleavage |
| NM_002872\|*RAC2* | 5 | N/A | 116-[AUGGCUGCAGAGCUUCGUUGA]-136 | Cleavage |
| NM_001005413\|*ZWINT* | 5 | N/A | 395-[AUGGAUCCAAAUGUUUGUUGA]-415 | Translation |
| NM_032997\|*ZWINT* | 5 | N/A | 586-[AUGGAUCCAAAUGUUUGUUGA]-606 | Translation |
| NM_152473\|*ERVV-1* | 5 | N/A | 980-[CGGGAGGCAGAGGUUGUGUUGA]-1001 | Cleavage |
| NM_172082\|*CAMK2B* | 5 | N/A | 1249-[GUCUGUGCAGGGGUUUACUGA]-1269 | Cleavage |
| NM_152556\|*C7orf60* | 5 | N/A | 105-[AGAAAUGCAGAGGUUUACAGA]-125 | Cleavage |
| NM_006766\|*KAT6A* | 5 | N/A | 1707-[UCAAAUGCACAGGUUUGUUGG]-1727 | Cleavage |
| NM_005347\|*HSPA5* | 5 | N/A | 856-[UAGGAUGUAAAGGUAUAUUGU]-876 | Cleavage |
| NM_033364\|*MAATS1* | 5 | N/A | 269-[GAGGAUGUGCUGGUUUGUUGA]-289 | Translation |
| NM_153218\|*LACC1* | 5 | N/A | 516-[AAAGAUGUAGAGGUUU-UUGA]-535 | Cleavage |
| NM_020123\|*TM9SF3* | 5 | N/A | 2235-[UAAGAUGUAG-GGUUUAUUGA]-2254 | Translation |
| NM_005406\|*ROCK1* | 5 | N/A | 1048-[AAAUAUUUAGAGGUUUGUUGG]-1068 | Cleavage |
| NM_013402\|*FADS1* | 5 | N/A | 1823-[ACCCAUGGAGAGGUUUGUCAA]-1843 | Cleavage |
| NM_000791\|*DHFR* | 5 | N/A | 381-[GUAUAUCCAGAGGUUUGUAGA]-401 | Cleavage |
| NM_176815\|*DHFRL1* | 5 | N/A | 383-[GUAUAUCCAGAGGUUUGUAGA]-403 | Cleavage |
| NM_014989\|*RIMS1* | 5 | N/A | 2175-[GGGGAGGCUGGGGUUUGUUGU]-2195 | Cleavage |

The cDNA library “*Homo sapiens* (human), transcript, Human genomic sequencing project” (available at psRNATarget server) was aligned with miR162 mature sequence (5'-UCGAUAAACCUCUGCAUCCAG-3'). N/A: not applicable.
